# Neurochemical underpinning of hemodynamic response to neuropsychiatric drugs: A meta- and cluster analysis of preclinical studies

**DOI:** 10.1177/0271678X20916003

**Published:** 2020-04-11

**Authors:** Lewis H Mervin, Ekaterina Mitricheva, Nikos K Logothetis, Angelo Bifone, Andreas Bender, Hamid R Noori

**Affiliations:** 1 Centre for Molecular Informatics, Department of Chemistry, University of Cambridge, Cambridge, UK; 2 Department of Physiology of Cognitive Processes, Max Planck Institute for Biological Cybernetics, Tübingen, Germany; 3 Imaging Science and Biomedical Engineering, University of Manchester, Manchester, UK; 4 Department of Molecular Biotechnology and Health Sciences, University of Torino, Torino, Italy; 5 Center for Neuroscience and Cognitive Systems, Istituto Italiano di Tecnologia, Rovereto, Italy; 6 McGovern Institute for Brain Research, Massachusetts Institute of Technology, Cambridge, MA, USA

**Keywords:** Pharmacological MRI, microdialysis, meta-analysis, rat brain

## Abstract

Functional magnetic resonance imaging (fMRI) is an extensively used method for the investigation of normal and pathological brain function. In particular, fMRI has been used to characterize spatiotemporal hemodynamic response to pharmacological challenges as a non-invasive readout of neuronal activity. However, the mechanisms underlying regional signal changes are yet unclear. In this study, we use a meta-analytic approach to converge data from microdialysis experiments with relative cerebral blood volume (rCBV) changes following acute administration of neuropsychiatric drugs in adult male rats. At whole-brain level, the functional response patterns show very weak correlation with neurochemical alterations, while for numerous brain areas a strong positive correlation with noradrenaline release exists. At a local scale of individual brain regions, the rCBV response to neurotransmitters is anatomically heterogeneous and, importantly, based on a complex interplay of different neurotransmitters that often exert opposing effects, thus providing a mechanism for regulating and fine tuning hemodynamic responses in specific regions.

## Introduction

Functional magnetic resonance imaging (MRI) is a commonly used non-invasive technology that allows mapping of brain activity at rest^1^ or in response to specific sensory stimuli, cognitive tasks^2^ or neuromodulatory and neurovascular activity induced by pharmacological agents;^3–9^ all by measuring spatiotemporal changes in blood flow, volume or blood oxygenation. In so-called pharmacological MRI (phMRI) experiments, acute administration of drugs induces local or global hemodynamic responses that can be detected with methods sensitive to different types of magnetization-relaxation processes yielding functional signals. While such functional MRI-signals (fMRI) can be causally linked to various types of neuronal and glial, e.g. astrocytic, activity,^10–12^ the actual, condition-specific neurochemical processes underlying the related phMRI signal changes remain elusive. Until now, two main strategies have been commonly pursued for gaining insights into the neurovascular mechanisms underlying phMRI responses. First, the dynamics of signals measured with different MRI methodologies were examined following local or systemic injection of various agonists and antagonists of neurotransmitters and neuromodulators;^13–21^ and second, phMRI experiments were combined with concurrent microdialysis,^7,21^ aiming to fathoming into the correlations between changes in neurotransmitter concentrations and the magnitude of alterations in cerebral blood circulation. Such studies reported similar time courses for changes in the functional signal and the substance-concentration measured by microdialysis.^7^ Yet microdialysis has limited spatial and temporal resolution, and most importantly, assessment of brain-wide neurochemical dynamics by means of such a minimally invasive method remains practically impossible.

**Figure 1.**
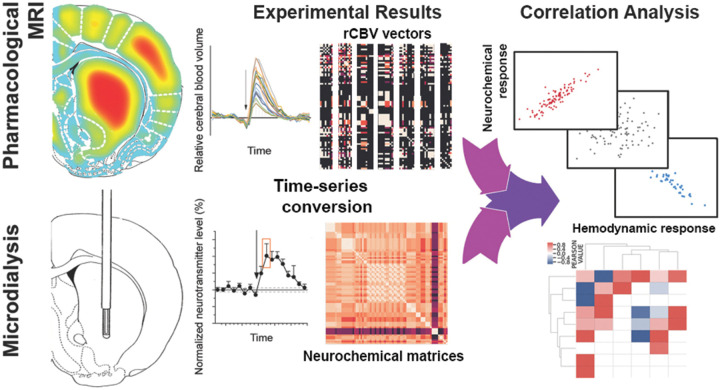
Schematic illustration of the convergence of spatiotemporal rCBV maps and microdialysis time-series to correlation matrices. In the first step, time-series for each observable type, namely phMRI and neurochemical data, are extracted from published studies. Each phMRI experiment provides rCBV time-series for 39 cortical and subcortical brain regions for baseline and post-injection phase. In turn, each in vivo microdialysis experiment provides trajectories of basal and drug-induced changes of neurotransmitter concentrations in only one brain region. As previously reported, measurements in up to 117 brain regions can be obtained. In order to reduce the data complexity and dimension, only the peak response and the time to peak from microdialysis experiments are extracted and converted into matrices for each transmitter with rows representing the brain regions and columns representing the drugs. In analogy, the peaks response and time were extracted from rCBV time-series. Subsequently, for rCBV experiments, the brain-wide response pattern to each drug is expressed by a normalized vector such that each component denotes the peak hemodynamic response in one brain region. Collectively, these vectors form a matrix that contains information on all drugs. The neurochemical brain-wide response pattern to each drug is expressed by a symmetric matrix with components denoting the response of a specific neurotransmitter within a specific brain region. In contrast to rCBV matrices that contain data from one experiment, the neurochemical matrices contain meta-analysed data of numerous microdialysis experiments. In the final step, a vector-matrix correlation analysis converges all data into information on similarity and differences of the two observable types.

In the present study, we report an alternative method that overcomes the latter problem by means of a mathematical-statistical, meta-analysis strategy. Specifically, while individual microdialysis experiments are restricted to measurements in single brain regions of interest (ROI), the number of studies, and thus the number of examined ROI, is vast. Therefore, microdialysis sessions collectively, if properly normalized, can converge into a whole-brain neurochemical map^22^ consistent with fMRI measurements. The aim of the present study has therefore been to achieve a data-convergence, by normalizing, summarizing and integrating phMRI and microdialysis data published studies in adult rats (Figure 1). Our analysis was conducted on phMRI and microdialysis time-series of brain response patterns to a selection of systemically administered neuropsychiatric drugs (amphetamine, apomorphine, cocaine, fluoxetine, nicotine, phencyclidine (PCP) and yohimbine). Our selection was prompted by the fact that these drugs represent different molecular modes of action, e.g. interactions with dopamine, acetylcholine, serotonin, noradrenaline, and glutamate systems, as well as discrete therapeutic/behavioral effects. Thereby, time-series of relative cerebral blood volume (rCBV) and the temporal profile of extracellular neurotransmitter concentrations with comparable sampling rates were extracted from published studies and transformed into multidimensional whole-brain response patterns, expressed by matrices of different size. Since microdialysis experiments often only provide data on changes of a limited number of transmitters in one brain region, an extensive meta-analysis was conducted to construct the whole-brain response matrix for each neurochemical system. By focusing on normalized peak response to drugs and a ROI-based averaging, we reduced data complexity and dimension. Subsequently, we developed and applied a meta-analysis-based approach, which enables us to calculate Pearson correlations of drug-induced peak hemodynamic response and peak neurotransmitter concentration changes at whole-brain level.

While numerous methods for analyzing the correlation of time-series of consistent sampling rate exist, analysis of datasets with differing spatiotemporal scale is a challenging problem, for which a generalized mathematical scheme is developed in this study. In addition, the influence of different variables, parameters and individual studies on overall findings was assessed by sensitivity analyses and demonstrates the robustness of the results.

Due to the spatial, morphological heterogeneity of the brain, different brain regions are likely to have distinct neurochemical underpinning of phMRI signals.^23^ To investigate whether and to what degree similarities and differences at ROI-level influence the whole-brain statistics and observations, we conducted secondary correlation and cluster analyses on a representative set (caudate putamen, globus pallidus, hippocampus, hypothalamus, nucleus accumbens, prefrontal cortex, septum and raphe nuclei) of cortical and subcortical brain areas. These brain regions play key roles within the mood, reward, and addiction neurocircuitries,^24^ which are known to be affected by the drugs under investigation in this study, particularly psychostimulants, antidepressants. Therefore, understanding the relationship between neurochemical and functional signals within these regions will not only improve our understanding of the hemodynamic response but may generate new hypotheses on the complex chemical interactions within brain networks.

## Methods

### Neurochemical dataset

As reported in our previous studies,^22,25–28^ drug-induced alterations in extracellular neurotransmitter concentrations as measured by in vivo microdialysis experiments in rats were extracted systematically and meta-analyzed to estimate averaged, normalized (i.e. baseline levels set to 100%) neurochemical response profiles. The matrices were obtained from Syphad database (www.syphad.com)^22^ for amphetamine, apomorphine, cocaine, fluoxetine, and nicotine. In analogy to data mining and integration steps of Syphad, the online portal of the National Library of Medicine (http://www.ncbi.nlm.nih.gov/pubmed/) including PubMed, PubMed Central and MEDLINE was used as the platform for literature research for phencyclidine and yohimbine. A systematic screening of the original research articles published until 01.01.2019 was performed based on the keywords rat (AND) microdialysis (AND) (brain region (OR) neurotransmitter (OR) metabolite (OR) neuropeptide) (AND) (phencyclidine (OR) PCP (OR) yohimbine). The keyword neurotransmitter is a general representative in the search string which was replaced by the actual name and/or abbreviation of transmitters and metabolites (e.g. dopamine, glutamate, homovanilic acid/HVA, etc.). In addition, the reference sections of identified papers as well as review and meta-analysis articles were then screened for further relevant citations. Only peer-reviewed original research articles in English language were chosen for data mining if they provided the absolute or relative change in neurotransmitter or metabolite concentrations within a brain region either numerically or in graphical manner. We excluded articles using animals other than rats. All selected studies were performed in outbred rats with no specific genotype or phenotype or provided data for a wild-type control group were included. Furthermore, animals did not receive any behavioral training prior to drug treatments. Abstracts and unpublished studies were not included.

In general, each microdialysis experiment provides time-series of basal concentration and drug-induced changes (in minute scale) for a limited number of neurotransmitters and metabolites within one brain region. These time-series are presented often as figures in the literature. Since microdialysis experiments are often conducted with different sampling rates, we only extract the peak response (x_i_) of a transmitter to the drug normalized to its baseline value and the time of the peak. In our previous studies,^22,25–28^ we conducted fixed-effect meta-analyses since the choice of fixed vs. random effect models depends strongly on the hypothesis as well as the extent of heterogeneity. In particular, if all included studies included in the analysis are reporting an estimate of the same effect and our goal is to calculate the overall effect size for the identified population without generalization to other populations, then a fixed effect model is more appropriate.^29,30^ In the present study, we compute random effect weighted averages for subgroups with minimized level of heterogeneity in experimental design, e.g. meta-analysis of the changes in a particular neurotransmitter such as glutamate in a specific brain region such as prefrontal cortex following a specific dosage of a drug. Due to the skewness of experimental designs towards male, adult, Sprague-Dawley rats, the expected level of heterogeneity is low. Nevertheless, the statistical heterogeneity can influence the findings and reduce our ability to draw robust overall conclusions. Thus, we calculated I^2^ which describes the proportion of total variation in study estimates due to heterogeneity,^31^ τ^2^ which denotes between-study heterogeneity and then calculated random-effect meta-analyses by integrating τ^2^ into the weights. To this end, we first calculated the weighted (using precisions w_i_ as weights) fixed effect average of study estimates X = ∑w_i_x_i_/∑w_i_, as introduced by Higgins and Thompson.^31^ We then conduct the Cochran Q-test of homogeneity, Q = ∑w_i_(x_i_-X).^2^ A moment-based estimate for τ^2^ is then given as τ^2^ = (Q-df)/(∑w_i_-(∑w_i_^2^_/_∑w_i_)), where df denotes degrees of freedom. Furthermore, I^2^ statistics is calculated by I^2^ = ((Q-df)/Q)×100%. While thresholds for the interpretation of I^2^ can be inaccurate, we follow Cochrane Handbook for Systematic Reviews of Interventions (https://handbook-5-1.cochrane.org/) and consider I^2^<40% as less important. The random effect weighted average is obtained by adding the between-study heterogeneity into the weights. Therefore, if τ^2^≪σ^2^ (variance of the study), then fixed and random effect models yield the same results, meaning that heterogeneity between studies does not affect the findings. This approach was further used to compute weighted averages of peak times (t_i_). Since the meta-analyses refer to different regions of interest, one may assume that the ROIs are provided (or nested) within studies, so that a hierarchical meta-analysis model would be more appropriate. However, less than 5% of all microdialysis studies provide information on several ROIs in a single report. Therefore, a hierarchical analysis was not applied.

Calculations were conducted in MATLAB R2015a. Results did not differ from estimates provided by metafor package in R.^32^ Codes, extracted data and results of random-effect meta-analyses and heterogeneity analyses are provided on the Center for Open Science platform (https://osf.io/zj6ae/).

With respect to concentration changes, the neurochemical matrix contains in each entry an average of normalized peak response of a particular neurotransmitter within a specific brain region. Thereby, amino acids, monoamines and their direct metabolites represent 95.2% of all matrix entries. Consequently, no statistically reliable conclusions can be made for other transmitter systems. Thus, the analysis was focused on response profiles of acetylcholine (ACh), dopamine (DA), γ-aminobutyric acid (GABA), glutamate (Glu), noradrenaline (NA), and serotonin (5-HT).

### Neurochemical response profiles

For each drug-dose combination, the response profile was presented as a numerical matrix with rows denoting the measured changes in neurotransmitter concentration and the columns presented brain areas within the neurochemical connectome of rat brain.^33^ In order to represent the data intuitively, i.e. decrease in concentration as a loss and increase as a gain in neurotransmitter concentration, data were converted by subtracting 100% from all data points. In other words, the drug effect on brain neurochemistry is reflected in the sign of the matrix components. As drugs were administered in different doses, numerous (*k*) matrices (R_ij_)^k^ per drug exist, whose elements refer to the change of a transmitter *i*, in brain region *j*.

Since, the matrices (R_ij_)^k^ contain the outcome of meta-analyses, i.e. average values of numerous microdialysis experiments, the maximum peak does not relate to outliers but to largest average values. To this end, we constructed a matrix M, such that each of its elements M_ij_ = max_k_(R_ij_)^k^ if (R_ij_)^k^>0 and M_ij_ =min_k_(R_ij_)^k^ if (R_ij_)^k^<0. An exploration of the temporal dynamics of drug-induced alterations of extracellular neurotransmitter dynamics as a function of drug dose (Supplementary Information, Table S1) outlines considerable differences between the both time-course of response and dose conditions across the assays for each compound. Therefore, a dynamical convergence of the matrices is not feasible.

The different spatial resolution of MRI and microdialysis experiments poses a further problem, as the anatomical ontologies are inconsistent. To overcome this issue, we converge the terminology of neurochemical connectome of rat brain^33^ and stereotaxic MRI template^19^ into a coarse-grained ontological framework analogous (Supplementary Information, Table S2) to our previous studies.^34^ The use of this ontology results in multiple cases of many-to-one mappings, for example, subregions of the ‘lateral hypothalamus’ and ‘medial hypothalamus’ are assigned to the (coarser) label of ‘hypothalamus’. In order to resolve this issue, a weighted ROI-average of neurotransmitter changes within all sub-regions was calculated using the numpy (https://numpy.org/) function ‘np.average’ in python, with the ‘weights’ parameter set to the number of rats. It is noteworthy that the heterogeneity in the cytoarchitecture often indicates that an averaging without considering anatomical properties is not optimal. However, the comparably small standard deviation of the matrix entries among sub-regions suggests that, from statistical point of view, the sub-region specific differences are negligible and averaging is admissible.

The resulting neurochemical response matrix (Supplementary Information, Figure S1) outlines how the seven drugs selected for this analysis comprise diverse neurochemical response patterns. For example, the prefrontal cortex and dopamine (CORTEX, PREFRONTAL and DA in the figure) show a mixture of both up or down response profiles across the six compounds profiled within this region. This is an intended consequence from the selection of diverse compounds for this work, since this enables the correlation of relative cerebral blood volume (rCBV) and neurochemical response across a variety of compound mechanism-of-actions (MOAs).

### phMRI dataset

Functional magnetic resonance imaging techniques enable the detection of hemodynamics (cerebral blood flow (CBF), cerebral blood volume (CBV) or oxygenation level of hemoglobin (BOLD)) response to changes in the activity of neuronal populations in proximity of blood vessels. For pharmacological stimuli, previous studies^35^ have shown a spatiotemporal coupling between the percent changes in CBV and BOLD relaxation rates. Nonetheless, CBV shows a higher contrast-to-noise ratio than BOLD, and was used as the detection method underlying the data used in this study. CBV experiments provide T_2_*- or T_2_-weighted image time series of transient drug-induced changes in blood volume with respect to a local resting state blood volume obtained by a contrast agent-enhanced relaxivity. The relative CBV (rCBV) is then obtained from the measured signal S(t), the estimated background signal in absence of the transient functional stimuli (B(t)) and the signal intensity prior to the administration of the contrast agent (S_pre_): *rCBV*(*t*)* = ln*(*S*(*t*)/*B*(*t*))/*ln*(*B*(*t*)/*S_pre_*). The peak response for different brain regions was extracted from the time-course of rCBV(t) that were made available by Angelo Bifone (IIT, Italy). The measurements have been previously processed and reported for 1 mg/kg D-amphetamine (*n* = 17) i.v.,^20^ 0.2 mg/kg apomorphine (*n* = 5) i.v.,^19^ 0.5 mg/kg cocaine (*n* = 8) i.v.,^21^ 10 mg/kg fluoxetine (*n* = 7) i.p.,^20^ 0.35 mg/kg nicotine (*n* = 9) i.v.,^18^ 0.5 mg/kg phencyclidine (*n* = 24) i.v.^16^ and 0.75 mg/kg yohimbine (*n* = 6) i.v.^17^

All animals used in the experiments were male adult Sprague-Dawley rats. Brain region designations were defined by the stereotaxic MRI template set for the rat brain^19^ and converged into the joint ontological framework (Supplementary Information, Table S2) as described above. Compound rCBV response was averaged into one value for each brain in the case of many-to-one ontology mappings (due to the nature of going from high to lower resolution), resulting into coarse-grained matrix for hemodynamic response (Supplementary Information, Figure S2).

### Neurochemical and MRI response profile correlation analysis

Compound neurochemical response profiles for each brain region-neurotransmitter pair as well as the corresponding brain-region specific rCBV responses were extracted. Pearson correlation was calculated between the two numerical arrays by using the scipy (https://www.scipy.org/) function scipy.stats.pearsonr in python. The output of this calculation ρ is a real number ranging from −1.0 (perfect negative correlation, i.e. rCBV changes are opposite to neurochemical alterations) to 1.0 (i.e. perfect correlation, where rCBV changes respond in an identical way to alterations of neurochemical levels). We consider ρ = 0 as no correlation, 0<|ρ|<0.2 as very weak, 0.2≤|ρ|<0.4 as weak, 0.4≤|ρ|<0.6 as moderate, 0.6≤|ρ|<0.8 as strong, and 0.8≤|ρ|<1 as very strong correlation^36^ indicating the strength of shared response between rCBV or neurochemical spaces upon compound administration. While confidence intervals of the correlations are provided, we report all correlation values including the non-significant relations as potential trends that may require further investigation in future studies. No individual study skewed the overall statistics; however, the inclusion and exclusion of specific drugs and/or neurotransmitter systems may affect the final findings. Thus, OFAT (one-factor-at-a-time) sensitivity analyses were performed a posteriori to ensure the robustness of the analysis results with respect to effect modifiers.

### Bi-cluster analysis of correlation results across brain region and neurotransmitters

Hierarchical bi-clustering of the correlation results was performed using the matrix of Pearson correlation values for the compounds across each neurotransmitter and brain region tuple, using the Seaborn ‘clustermap’ function with the method set to ‘average’, and the metric set to ‘Euclidean’.

## Results

The keyword-based search of abstracts, titles, or both identified 2449 publications. Out of these, 774 studies were original research articles on in vivo microdialysis in 22,489 rat brains and provided data for neurochemical meta-analysis. The phMRI studies and the majority microdialysis experiments were conducted in adult (89.1%), male (90.5%), Sprague-Dawley rats (67.9%), while there were variations in other experimental parameters used in microdialysis studies (Supplementary Information, Figure S3). In contrast to phMRI studies, 88.7% of microdialysis experiments (*n* = 20,383) were conducted in awake, freely moving animals. Thereby, the choice of anesthetic agents and the applied concentrations varied notably (Supplementary Information, Table S3). The heterogeneity analyses suggest that in 20% of cases the I^2^ was larger than 40% and the fixed- and random effect model results differed. Nevertheless, only 2.5% of such cases were associated with potential effect modifiers such as age, strain and use of anesthesia. For instance, administration of 10 mg/kg cocaine increased dopamine concentration in adolescent animals by 306 ± 3% and thus, significantly less (*p *<* *0.05, one-way ANOVA) than in adult rats (371 ± 0.2%).

Heterogeneity tests further suggest that the application of anesthesia affects the striatal dopaminergic response to 10 mg/kg of phencyclidine. While data obtained from awake, freely moving animals suggest study homogeneity (τ^2^ = 0.02; I^2^ = 33%; random-effect mean: 252% with 95% confidence interval: [234, 270]), the application of anesthesia is associated with significant (*p* < 0.00001) heterogeneity (τ^2^ = 0.27; I^2^ = 94%; random-effect mean: 232% with 95% confidence interval: [173, 292]). Therefore, in spite of few exceptions, the analyses reported in this study remain largely robust with respect to experimental parameters.

### Common features of neurochemical response

We extracted and conducted meta-analysis on percentage changes in neurotransmitter (i.e. acetylcholine (ACh), 5-HT, dopamine (DA), GABA, glutamate (Glu), and noradrenaline (NA)) concentrations relative to basal levels within 18 cortical and subcortical brain areas (amygdala, anterior cingulate cortex, bed nucleus of stria terminalis, caudate putamen, frontal cortex, globus pallidus, hippocampus, hypothalamus, nucleus accumbens, prefrontal cortex, posterior cingulate cortex, raphe, septum, substantia nigra, temporal cortex, ventral pallidum, and ventral tegmental area). The drugs under consideration were applied systematically in wide ranges of doses: amphetamine (0.2– 30 mg/kg), apomorphine (0.1–3 mg/kg), cocaine (0.05–40 mg/kg), fluoxetine (0.3–40 mg/kg), nicotine (0.05–135 mg/kg), phencyclidine (0.3–20 mg/kg) and yohimbine (0.1–5 mg/kg). The heterogeneity tests of the in vivo microdialysis experiments suggest that the study estimates are largely homogeneous (median τ^2^ = 0.02; median I^2^ = 27.61%). However, 18.3% of the cases showed considerable heterogeneity (i.e. 75% <I^2^ < 100%) that justifies the choice of random-effect model for meta-analysis.

Thereby, the results suggest diverse dynamical patterns for different neurotransmitter systems, which show different levels of sensitivity to specific molecular modes of action of the drugs. All drugs in all doses increase 5-HT and ACh levels in all investigate brain areas by approximately 247 ± 30% and 188 ± 11%. However, apomorphine in the range of 0.3–3 mg/kg moderately reduced ACh levels in caudate putamen from 10 to 25%. Moreover, apomorphine reduced dopamine levels in caudate putamen, nucleus accumbens and prefrontal cortex by up to 73%. All other drug-dose combinations increase dopamine levels notably in cortical and subcortical brain regions (in average 464 ± 41%). GABA dynamics represents a non-linear response pattern, that timing but not in magnitude, is opposite to glutamatergic response. In particular, low concentrations of nicotine and apomorphine reduce GABA levels in subcortical structures such as hypothalamus and ventral pallidum, and in prefrontal cortex whereas phencyclidine and amphetamine and higher concentrations of apomorphine enhance GABA concentrations in globus pallidus (∼160%), caudate putamen (∼187%), septum (∼210%), and prefrontal cortex (∼ 200%). Glutamate in turn is increased by all drugs by approximately 77 ± 14%, but 10 mg/kg of fluoxetine which reduces Glu levels in frontal cortex by 40%. NA as well is enhanced by all drugs by in average 245 ± 76%.

### Common features of hemodynamic response

As previously reported,^3,6,16–20^ hemodynamic response to drug challenges was associated with selective activation of pathways based on their molecular modes of action and in a dose-dependent manner. These findings will be briefly summarized in the following. While amphetamine induces an anatomically indistinctive activation of the brain, the response to other drugs including psychostimulants such as cocaine and phencyclidine was specific. In particular, cocaine injection leads to the significant activation of various cortical (such as prefrontal, frontal, and cingulate) and subcortical (caudate putamen, nucleus accumbens, and thalamus) areas that are associated with reward circuitry. Significant positive rCBV changes (*Z *>* *2.3 vs. vehicle, cluster correction at *p * = * *0.05) induced by phencyclidine were observed in cortico-limbo-thalamic regions (medial prefrontal, cingulate, orbito-frontal, and retrosplenial cortices), with extension into the motor, visual, parietal-, and temporal association and rhinal cortices. Significant activation was further observed in subcortical brain regions such as extended amygdala, thalamus and postero-dorsal, antero-dorsal and ventral and posterior hippocampus. Fluoxetine, in turn, shows a largely cortical effect as well as foci in the hippocampus, ventral tegmental area and in the thalamus.^20^ Similarly, yohimbine administration leads to the activations in several cortico-limbic areas (*Z *>* *2.3, *p*_c_<0.01) such as prefrontal, cingulate, orbito-frontal, and retrosplenial cortex, with extension into the motor, visual, parietal-, and temporal-association areas. Moreover, in addition to extra-hypothalamic stress circuit (e.g. extended amygdala), hemodynamic response in subcortical structures such as caudate putamen, nucleus accumbens, ventromedial and dorsolateral thalamus, and ventral hippocampal area was significantly increased. The administration of either apomorphine or nicotine induces significant activation in particularly strong effects in the orbitofrontal and prefrontal cortices, in the insular cortex extending back to the ectorhinal/perirhinal cortices and thalamus. Nicotine further enhances rCBV in the amygdala, ventrocaudal hippocampal structures, and nucleus accumbens. Taken together, the overall phMRI findings indicate largely overlapping cortical (prefrontal cortex) and subcortical (Hippocampus, hypothalamus, nucleus accumbens, septum, caudate putamen and ventral tegmental area) hemodynamic response patterns due to the action of these neuropsychiatric drugs under investigation.

### Correlation analyses

The analysis of similarities between hemodynamic response and changes of neurotransmitter concentrations induced by individual drugs at whole-brain level suggest very strong correlations of 5-HT (ρ_5-HT_ = 0.88) and noradrenaline (ρ_NA_ = 0.81) for nicotine, 5-HT (ρ_5-HT_ = 0.82) and GABA (ρ_GABA_ = −0.99) for phencyclidine and moderate negative correlations of ACh (ρ_ACh_ = −0.48) and 5-HT (ρ_5-HT_ = −0.43) for amphetamine. For other drugs, i.e. apomorphine, cocaine, fluoxetine and yohimbine, neurochemistry either very weakly or weakly correlates with rCBV, and thus no meaningful conclusion can be drawn.

In order to discover common principles, we calculated how neurochemical and functional response patterns correlate for the set of all drugs. The analysis suggests that changes in cerebral blood volume, while relating to penetrating arteries and cortical layers,^37^ correlate only very weakly with changes in neurotransmitter concentration as measured by microdialysis at whole-brain level (ρ_DA_ = 0.089, ρ_Glu_ = 0.088, ρ_NA_ =  0.043, ρ_GABA_ = 0.025, ρ_ACh_ = 0.010, ρ_5-HT_ = −0.101). Interestingly, the sensitivity analyses (Supplementary Information, Tables S4 and S5) suggest that the exclusion of each individual drug, but amphetamine, does not alter the statistical findings. Excluding amphetamine from the set of drugs leads to strong positive correlations of hemodynamic response with noradrenaline (ρ_NA_ = 0.60), and strong negative correlation with GABA (ρ_GABA_ = −0.64). It is noteworthy that amphetamine increases noradrenaline concentrations and rCBV response in all brain regions, the response magnitude at whole-brain level is negatively correlated (ρ_NA_ = −0.33), which may result into a diminishing correlation of NA and hemodynamic response due to an individual drug (Supplementary Information, Table S6).

**Figure 2.**
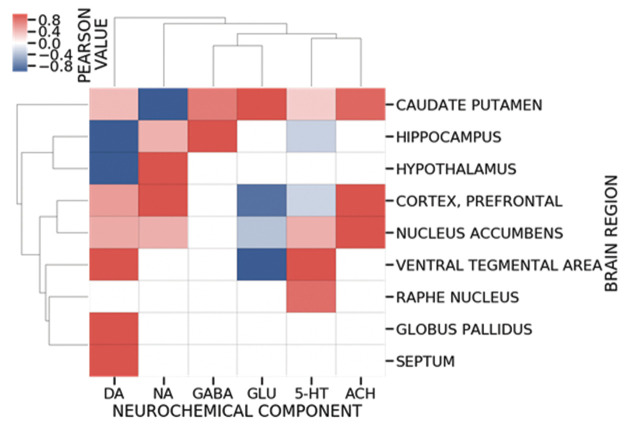
Region of interest (ROI)-based Pearson correlation between drug-induced neurochemical and hemodynamic response patterns shows an anatomical heterogeneity underlying the relationship between neurotransmitter concentrations and rCBV signal changes. The complex and region-specific interplays of different neurotransmitter systems generate hypotheses on internal functioning of different neurocircuitries in the brain. For instance, the push-pull action within VTA (positive for dopamine and 5-HT, negative for glutamate) appears to play a role in the regulation of hemodynamic response and the functional control of reward circuitry. Correlation values were calculated if data were provided for at least two of the seven drugs under consideration. Negative and positive correlations are presented as shades of blue and red, respectively.

The lack of even moderate correlation of neurotransmitter and rCBV response to drugs at global, whole-brain level may further be due to spatial heterogeneity in terms of differences in neurovasculature, receptor distributions and cytoarchitecture of different brain areas. To evaluate the impact of spatial structural variability, we conducted a ROI-based correlation analysis (Supplementary Information, Figures S4 and S5). As previously reported,^22^ there is a general skewness of microdialysis studies by means of an overemphasis on monoamine systems (particularly dopamine) within the reward circuitry, which is also reflected in the neurochemical database. The missing values related to other neurotransmitter systems and brain regions do not permit a complete correlation analysis for all regions of interest. In other cases, if data were provided for at least two of the seven drugs under consideration, then Pearson correlations of neurochemical and rCBV responses were calculated (Figure 2). While this strategy allows us to conduct correlation analysis, the obtained correlation values directly relate to the number of drugs contributing to the calculation. Thereby, smaller numbers of drugs generally lead to higher magnitudes of correlations. Therefore, the findings are presented as pairs of Pearson correlation in brain region A for neurotransmitter B, ρ_A_^B^, and number of drugs included in the analysis *m*, to avoid an over-interpretation of our findings (Supplementary Information, Table S7). All brain areas but caudate putamen show a positive correlation of noradrenaline transmission and a negative correlation of glutamate dynamics with hemodynamic response. In prefrontal cortex, alterations in ACh and NA correlate perfectly with changes in rCBV (ρ_PFC_^ACh^ = ρ_PFC_^ACh^ = 1, m = 2), dopamine correlates moderately (ρ_PFC_^DA^ = 0.57, m = 4), whereas 5-HT and glutamate are negatively correlated to hemodynamic response (ρ_PFC_^5-HT^ =−0.27, m = 4; ρ_PFC_^Glu^ = −0.86, m = 3). In hippocampus, GABA and NA changes correlate positively with functional response (ρ_HC_^GABA^ = 1, m = 2; ρ_HC_^NA^ =0.46, m = 3), 5-HT and dopamine changes correlate negatively with rCBV changes (ρ_HC_^5-HT^ = −0.29, m = 3; ρ_HC_^DA^ = −1, m = 2) and correlations between changes in acetylcholine concentrations and rCBV are negligible (ρ_HC_^ACh^ = −0.007, m = 4). Dopamine dynamics perfectly correlates positively and negatively with hemodynamic response in globus pallidus, septum and hypothalamus, respectively (ρ_GP_^DA^ = 1, m = 2; ρ_Septum_^DA^ = 1, m = 2; ρ_HyT_^DA^ = −1, m = 2), while noradrenaline changes in hypothalamus positively correlate with rCBV response (ρ_HyT_^NA^ = 1, m = 2). In raphe, drug-induced alterations in 5-HT levels are very strongly correlated to hemodynamic response (ρ_Raphe_^5-HT^ = 0.85, m = 3). Dopamine and 5-HT concentrations in the ventral tegmental area correlate with rCBV changes perfectly (ρ_VTA_^DA^ = 1, m = 2; ρ_VTA_^5-HT^ = 1, m = 2), while glutamate system shows the opposite behavior (ρ_VTA_^Glu^ = −1, m = 2). A similar behavior can be observed in nucleus accumbens, where all transmitters but glutamate are positively correlated to changes in hemodynamic response (ρ_Acb_^ACh^ = 1, m = 2; ρ_Acb_^NA^ = 0.59, m = 3; ρ_Acb_^DA^ = 0.51, m = 6; ρ_Acb_^5-HT^ = 0.51, m = 5; ρ_Acb_^Glu^ = −0.33, m = 4). In caudate putamen, all transmitter but noradrenaline correlate positively with rCBV changes (ρ_CPu_^Glu^ = 1, m = 2; ρ_CPu_^ACh^ = 0.89, m = 3; ρ_CPu_^DA^ = 0.78, m = 6; ρ_CPu_^GABA^ = 0.77, m = 3; ρ_CPu_^5-HT^ = 0.29, m = 4; ρ_CPu_^NA^ = −1, m = 2). It is noteworthy that m = 2 without an exception leads to perfect positive and negative correlation scores, indicating that it may not present an appropriate choice for discovering true patterns. In contrast, for m ≥ 3, correlation patterns emerge that show robustness with respect to brain areas. In average, drug-induced changes in glutamate levels appear negatively correlated with rCBV, whereas noradrenaline and dopamine systems are positively correlated with relative cerebral blood volume alterations. Thereby, 5-HT reveals a diverse response pattern, as it positively correlates with hemodynamic response in nucleus accumbens and raphe and negative correlations in prefrontal cortex and hippocampus, which may relate to serotoninergic projections in rat brain originating in raphe and strongly innervating nucleus accumbens with a weaker outreach of cortical regions.^33^ While the rough sampling times of microdialysis experiments and differences in measurement time-scales with phMRI experiments did not allow a quantitative comparison of drug response trajectories, we analyzed peak response times (Supplementary Information, Table S8) to include the temporal dimension in our investigation. Thereby, the response time is negatively (Supplementary Information, Figure S6), yet not significantly (overall *p* = 0.57) correlated with the hemodynamic response for most neurotransmitters. In agreement with previous studies,^38^ glutamate and GABA show an opposite dynamics, i.e. the Pearson coefficients for glutamate and GABA are of opposite sign.

## Discussion

fMRI is widely used to study the operational organization of the brain. The exact relationship between the measured functional signal and the underlying neural activity, however, remains unclear. Previous studies^11^ have examined the relationship between mesoscopic electrical signals (e.g. various frequency ranges of the mean extracellular field potential, often dubbed as Local Field Potentials, LFP) and neurovascular activity patterns, such as those reflecting changes in blood oxygenation. Extending this methodology by using multishank and multisite electrodes can also provide global neuronal and vascular activity patterns that may help interpreting brain states. However, a concurrent widespread mapping of the regional neurochemical processes reflecting the activity patterns induced, for example, by neuromodulators, is currently impossible, despite of its utmost importance for drug design and development. The gap is mainly due to the experimental limitations of neurochemical measurement techniques by biosensors or in vivo microdialysis. While these methods allow the observation of drug-induced alterations of extracellular neurotransmitter concentrations at a small spatiotemporal scale, corresponding to a small number of voxels in the usual fMRI studies, simultaneous observation of drug effects on the entire brain is not possible, as the minimally invasive methods such as microdialysis – beyond their limited spatiotemporal resolution – have also restricted regional extent of sampling. Pharmacological MRI (phMRI) could – in principle – overcome the latter limitation, if local changes in the phMRI signals are consistently related to the concentration changes of different neuromodulators or drugs quantified by means of biosensors. In other words, a robust, potentially causal relationship between chemicals and phMRI signal changes, eventually permitting a linear (or non-linear) estimation of an impulse response function for various regions, which in turn allows the inverse estimation of local neurochemical changes by measuring the phMRI signal-changes could indeed permit a reliable convergence of regional data. Targeted pharmacological manipulation of neurotransmitter systems and simultaneous measurement of hemodynamic response has indeed already provided some insights into the effect of drugs on neurovascular coupling.^3,5,6,13^ Puzzling, however is the fact that the changes in the fMRI signal are often induced by alterations in numerous dynamically coupled neurotransmitter systems that respond directly (by the drug acting on various proteins with different affinity) or indirectly (i.e. through network interactions) to the administration of neuropsychiatric drugs. While such sets of neurochemical systems are non-unique and may differ among drugs, the conjunction set (i.e. the ensemble of neurotransmitters associated with hemodynamic response that react non-specifically to all drugs) is unique and is critical to characterize the link between neurochemical and functional dynamics.

Using a set of representative neuropsychiatric drugs, we were indeed able to find the above-mentioned conjunction set as a universal neurochemical underpinning of hemodynamic response. Notably, our whole-brain correlation analysis did not directly lead to the identification of sets of neurotransmitters associated with functional signal changes. However, our sensitivity analyses suggest that the null-result is mostly due to two factors, namely the small number of drugs for which data exists and large variance in how different brain regions respond to drugs. Based on currently existing data, the robust core of our findings suggests a positive correlation of noradrenaline and a positive correlation of glutamatergic dynamics with hemodynamic response. This finding is in agreement with previous studies that have shown direct noradrenergic input to cerebral penetrating arteries and intracortical microvessels, indicating a direct role of noradrenaline in blood–brain barrier and cerebral blood flow.^39–41^

Surprisingly, our analysis does not provide evidence that the magnitude of glutamate changes is driving the functional response (if anything, an anticorrelation was found). However, in agreement with recent functional magnetic resonance spectroscopy (fMRS)^38^ and combined fMRI-MRS^42^ in humans, the time courses of changes in glutamate and BOLD signals induced by visual stimulation are positively correlated. Our findings are further consistent with an opposite dynamics of glutamate and GABA response to stimuli.^38^ In addition, a feasible and clear indication of the implication of the noradrenergic system is identified. Both, glutamate and noradrenaline, are well-known to play key roles in neurovascular coupling, for instance via the regulation of astroglial function and cerebral microvascular^4,8,39,43–45^ and thus, the intensity of fMRI signals.

While inhibition of metabotropic glutamate receptor generally blocks the vascular response, their activation by agonists leads to enhancements of intracellular Ca2+ concentration in astrocytes and consequently vasodilation as induced by neuronal stimulation.^45^ Furthermore, simultaneous multimodal measurements of neuronal activity and glutamate concentration in thalamus indicate a positive correlation with functional signals.^46^ However, the importance of astrocytes in regulation of cerebral blood flow remains unresolved.^47,48^

We speculate that our inability to discover a positive correlation between the magnitude of glutamate-rCBV correlations is due to two reasons. Glutamate is the main excitatory neurotransmitter in the central nervous system that is metabolized and released by neurons and also astrocytes.^49–51^ Thereby, the astrocytes not only regulate the glutamate levels but the dynamics of this process may prevent observation of refined glutamatergic oscillations. Therefore, the rough time-resolution of microdialysis experiments (in minutes) leads to not only sampling active glutamate molecules associated with neurotransmission but also to an added concentration of free glutamate by astrocytes. This, in turn, affects the correlation analysis directly and may overshadow actual links between changes in glutamate concentration and hemodynamic response.

However, the sign of the correlation may also relate to an anatomical gradient between cortical and subcortical regions, as neural activity appears to positively correlate with functional activity for cortical and negatively for subcortical regions.^52^ Indeed, previous studies have shown a high level of anatomical heterogeneity in neurovasculature,^12,53,54^ receptor distribution^33^ and generally BOLD signal among different brain regions^52^ that may have led to positive correlations in some and negative correlations in other brain areas and subsequently a net-zero (meaningless) correlation at whole-brain level. Nonetheless, the findings are based entirely on correlations without an exploration of confidence intervals and statistical significance. With growing number of experiments and data, the investigation needs to be complemented by such analyses to ensure robustness of the identified correlations.

Beyond the global observations, our ROI-based analysis further suggests that the involvement of neurotransmitter systems in hemodynamic response is anatomically heterogeneous and that not a single neurochemical mechanism but rather a complex interplay of various transmitter systems contributes to neurovascular coupling. The correlation of rCBV changes with neurochemistry in the main source of dopaminergic projections within the reward circuitry, namely ventral tegmental area (VTA), is an example of this observation. Our findings suggest that a push-pull action (positive for dopamine and 5-HT, negative for glutamate) is responsible for the regulation of hemodynamic response and the functional control of this important brain region. Such complex and region-specific interplays of different neurotransmitter systems can also be used to improve our understanding and generate hypotheses on internal functioning of different neurocircuitries in the brain. For instance, a plausible observation is that dopamine correlates positively with rCBV within the mesolimbic system and exerts opposite effects in other regions.

While our results are feasible in light of previous experimental findings, our study and its conclusions are intrinsically limited to the databases they rely on. phMRI measurements provide global, whole-brain observations; however, phMRI experiments have only been conducted using a small set of neuropsychiatric drugs. The opposite is true for microdialysis experiments that provide data for at least 260 drugs,^22^ however often incomplete with respect to number of measured brain regions and transmitter systems. Furthermore, due to differences in time-scales of measurements, the presented analysis is only based on peak response (i.e. peak response magnitude and time) and not on spatiotemporal trajectories, which may contain critical information. In general, trajectories of dynamical systems depend on initial values/conditions and a proper analysis needs to take both the initial values and temporal dynamics into consideration. For instance, previous MRS investigations^38^ indicate that different baseline conditions may explain diverging signal changes of GABA and glutamate. In the included microdialysis experiments, the animals were wild-type, drug-naïve and did not undergo any behavioral manipulations (e.g. stress) prior to the measurements. Thus, one can safely assume that the baseline conditions are comparable. To our surprise, numerous studies do not report the basal concentrations. For example, 10 out of 15 studies on Yohimbine and over 50% of studies on PCP do not report basal values. Thus, if we set it as a key feature of the analysis, then numerous studies would have been excluded. In addition, it is noteworthy that the quantity of analyte collected by microdialysis (i.e. absolute concentration) often represents only a fraction of the ‘true’ extracellular neurotransmitter levels. Differences in concentrations of calcium (mM) of perfusate, flow rate (µl/min) or membrane surface area (often reported as membrane length (mm) and membrane outer-diameter (µm)) affect the relative recovery, i.e. the ratio between the actual extracellular concentration of an analyte and its dialysate concentration, significantly.^55^ To avoid this issue, a large number of microdialysis studies on drug effects normalize the drug-induced response to the basal levels measured in the study and report exclusively the normalized (% baseline) values. Since rCBV, as well, represents a relative measure of drug response, the comparison of the two variables is feasible.

A further limitation of our study is due to the fact that phMRI experiments are conducted in anesthetized animals, whereas the majority of microdialysis experiments were performed in awake, freely moving rats. While drug–drug interactions generally may influence the findings in a critical manner, analysis of variance of microdialysis data obtained from awake and anesthetized rats for the drugs under consideration did not show any significant differences between the groups. However, the application of halothane may induce notable heterogeneity in measurements of striatal dopamine concentrations following acute administration of cocaine. While halothane is not a commonly used anesthetic agent in the context of functional neuroimaging, depending on the applied anesthesia, the outcome of phMRI may vary. Therefore, a more accurate comparison of phMRI and microdialysis experiments would benefit from phMRI data obtained from awake animals.

## Conclusions

Our findings suggest that the neurochemical basis of changes in functional MRI signals most likely relates to a dynamical interplay of glutamate and noradrenergic transmission in the brain. However, more phMRI data on preferably awake animals are required to detect the refined role of other transmitter systems such as dopamine, 5-HT and GABA in regulating the magnitude of functional signal changes.

## Supplemental Material

JCB916003 Supplementary Information - Supplemental material for Neurochemical underpinning of hemodynamic response to neuropsychiatric drugs: A meta- and cluster analysis of preclinical studies Click here for additional data file. Supplemental material, JCB916003 Supplementary Information for Neurochemical underpinning of hemodynamic response to neuropsychiatric drugs: A meta- and cluster analysis of preclinical studies by Lewis H Mervin, Ekaterina Mitricheva, Nikos K Logothetis, Angelo Bifone, Andreas Bender and Hamid R Noori in Journal of Cerebral Blood Flow & Metabolism
