# Supplementary material for: Neurochemical underpinning of hemodynamic response to neuropsychiatric drugs: A meta- and cluster analysis of preclinical studies
Source: J Cereb Blood Flow Metab. 2020 Apr 11;41(4):874–85. doi: 10.1177/0271678X20916003 (PMC7983335; doi:10.1177/0271678X20916003)
Supplement: JCB916003 Supplementary Information - Supplemental material for Neurochemical underpinning of hemodynamic response to neuropsychiatric drugs: A meta- and cluster analysis of preclinical studies [file JCB916003_Supplementary_Information.pdf]

# Supplementary Information

## Neurochemical underpinning of hemodynamic response to neuropsychiatric drugs: A meta- and cluster analysis of preclinical studies

Lewis H. Mervin[1], Ekaterina Mitricheva[2], Nikos K. Logothetis[2,3], Angelo Bifone[4,5], Andreas Bender[1], Hamid R. Noori[2,6*]

[1] Centre for Molecular Informatics, Department of Chemistry, University of Cambridge, CB2 1EW, Cambridge UK

[2] Department of Physiology of Cognitive Processes, Max Planck Institute for Biological Cybernetics, 72076 Tübingen, Germany

[3] Imaging Science and Biomedical Engineering, University of Manchester, M13 9PL Manchester, United Kingdom

[4] Department of Molecular Biotechnology and Health Sciences, University of Torino, 10126, Torino, Italy

[5] Center for Neuroscience and Cognitive Systems, Istituto Italiano di Tecnologia, 38068 Rovereto, Italy

[6] McGovern Institute for Brain Research, Massachusetts Institute of Technology, Cambridge, MA 02139, Unites States

*Corresponding Authors:

Hamid R. Noori, Max Planck Institute for Biological Cybernetics, Max-Planck-Ring 8, 72076 Tübingen, Germany. E-mail:

hamid.noori@tuebingen.mpg.de ; Phone: +49 (7071) 601-1710

## List of references included in the phencyclidine and yohimbine neurochemical analyses[1]

PHENCYCLIDINE


Abekawa, T., et al. (2003). "Effects of NRA0045, a novel potent antagonist at dopamine D4, 5-HT2A, and alpha1 adrenaline receptors, and NRA0160, a selective D4 receptor antagonist, on phencyclidine-induced behavior and glutamate release in rats." Psychopharmacology (Berl) **169**(3-4): 247-256.

Abekawa, T., et al. (2006). "Role of the simultaneous enhancement of NMDA and dopamine D1 receptor-mediated neurotransmission in the effects of clozapine on phencyclidine-induced acute increases in glutamate levels in the rat medial prefrontal cortex." Naunyn Schmiedebergs Arch Pharmacol **374**(3): 177-193.

Adams, B. and B. Moghaddam (1998). "Corticolimbic dopamine neurotransmission is temporally dissociated from the cognitive and locomotor effects of phencyclidine." J Neurosci **18**(14): 5545-5554.

Amargos-Bosch, M., et al. (2006). "Clozapine and olanzapine, but not haloperidol, suppress serotonin efflux in the medial prefrontal cortex elicited by phencyclidine and ketamine." Int J Neuropsychopharmacol **9**(5): 565-573.

Amitai, N., et al. (2012). "Repeated phencyclidine administration alters glutamate release and decreases GABA markers in the prefrontal cortex of rats." Neuropharmacology **62**(3): 1422-1431.

Carboni, E., et al. (1989). "Amphetamine, cocaine, phencyclidine and nomifensine increase extracellular dopamine concentrations preferentially in the nucleus accumbens of freely moving rats." Neuroscience **28**(3): 653-661.

Chapman, C. D., et al. (1990). "Effects of phencyclidine on extracellular levels of dopamine, dihydroxyphenylacetic acid and homovanillic acid in conscious and anesthetized rats." Neuropharmacology **29**(4): 319-325.

Etou, K., et al. (1998). "Ceruletide inhibits phencyclidine-induced dopamine and serotonin release in rat prefrontal cortex." Pharmacol Biochem Behav **61**(4): 427-434.

Greenslade, R. G. and S. N. Mitchell (2004). "Selective action of (-)-2-oxa-4-aminobicyclo[3.1.0]hexane-4,6-dicarboxylate (LY379268), a group II metabotropic glutamate receptor agonist, on basal and phencyclidine-induced dopamine release in the nucleus accumbens shell." Neuropharmacology **47**(1): 1-8.

Hertel, P., et al. (1995). "Effects of D-amphetamine and phencyclidine on behavior and extracellular concentrations of neurotensin and dopamine in the ventral striatum and the medial prefrontal cortex of the rat." Behav Brain Res **72**(1-2): 103-114.

Hondo, H., et al. (1995). "The effect of phencyclidine on the basal and high potassium evoked extracellular GABA levels in the striatum of freely-moving rats: an in vivo microdialysis study." Brain Res **671**(1): 54-62.

Hondo, H., et al. (1994). "Effect of phencyclidine on dopamine release in the rat prefrontal cortex; an in vivo microdialysis study." Brain Res **633**(1-2): 337-342.

Kehr, J., et al. (2018). "Effects of cariprazine on extracellular levels of glutamate, GABA, dopamine, noradrenaline and serotonin in the medial prefrontal cortex in the rat phencyclidine model of schizophrenia studied by microdialysis and simultaneous recordings of locomotor activity." Psychopharmacology (Berl) **235**(5): 1593-1607.


---

[1] The focus of this study is the correlation of changes in amino acid and monoamine concentrations with rCBV following drug administration. Nonetheless, in process of exploring potential relationships with other neurotransmitters and metabolites, we collected further relevant data that has been made available open-access via www.syphad.com and https://osf.io/zj6ae/.

Kuroki, T., et al. (1999). "Effects of the serotonin2A/2C receptor agonist and antagonist on phencyclidine-induced dopamine release in rat medial prefrontal cortex." Prog Neuropsychopharmacol Biol Psychiatry **23**(7): 1259-1275.

Li, Z., et al. (2010). "The novel neurotensin analog NT69L blocks phencyclidine (PCP)-induced increases in locomotor activity and PCP-induced increases in monoamine and amino acids levels in the medial prefrontal cortex." Brain Res **1311**: 28-36.

Lillrank, S. M., et al. (1994). "Systemic phencyclidine administration is associated with increased dopamine, GABA, and 5-HIAA levels in the dorsolateral striatum of conscious rats: an in vivo microdialysis study." J Neural Transm Gen Sect **95**(2): 145-155.

Maeda, J., et al. (2003). "Different roles of group I and group II metabotropic glutamate receptors on phencyclidine-induced dopamine release in the rat prefrontal cortex." Neurosci Lett **336**(3): 171-174.

Martin, P., et al. (1998). "Systemic PCP treatment elevates brain extracellular 5-HT: a microdialysis study in awake rats." Neuroreport **9**(13): 2985-2988.

Mathe, A. A., et al. (1996). "The psychotomimetic drugs D-amphetamine and phencyclidine release calcitonin gene-related peptide in the limbic forebrain of the rat." J Neurosci Res **46**(3): 316-323.

*Mele, A., et al. (1997). "Alterations in striatal dopamine overflow during rotational behavior induced by amphetamine, phencyclidine, and MK-801." Synapse **26**(3): 218-224.

Mele, A., et al. (1998). "The role of striatal dopaminergic mechanisms in rotational behavior induced by phencyclidine and phencyclidine-like drugs." Psychopharmacology (Berl) **135**(2): 107-118.

Millan, M. J., et al. (1999). "Contrasting mechanisms of action and sensitivity to antipsychotics of phencyclidine versus amphetamine: importance of nucleus accumbens 5-HT2A sites for PCP-induced locomotion in the rat." Eur J Neurosci **11**(12): 4419-4432.

Nishijima, K., et al. (1996). "Differential effects of phencyclidine and methamphetamine on dopamine metabolism in rat frontal cortex and striatum as revealed by in vivo dialysis." Synapse **22**(4): 304-312.

Paasonen, J., et al. (2017). "Dose-response effect of acute phencyclidine on functional connectivity and dopamine levels, and their association with schizophrenia-like symptom classes in rat." Neuropharmacology **119**: 15-25.

Pickering, C., et al. (2010). "Ethanol and phencyclidine interact with respect to nucleus accumbens dopamine release: differential effects of administration order and pretreatment protocol." Front Behav Neurosci **4**: 32.

Schiffer, W. K., et al. (2001). "Gamma vinyl-GABA differentially modulates NMDA antagonist-induced increases in mesocortical versus mesolimbic DA transmission." Neuropsychopharmacology **25**(5): 704-712.

Steinpreis, R. E. and J. D. Salamone (1993). "The role of nucleus accumbens dopamine in the neurochemical and behavioral effects of phencyclidine: a microdialysis and behavioral study." Brain Res **612**(1-2): 263-270.

Takahashi, S., et al. (2001). "MS-377, a novel selective sigma(1) receptor ligand, reverses phencyclidine-induced release of dopamine and serotonin in rat brain." Eur J Pharmacol **427**(3): 211-219.

* Data provided in Mele, A. et al. (1998). Redundancy avoided.

YOHIMBINE

Abercrombie, E. D., et al. (1988). "Characterization of hippocampal norepinephrine release as measured by microdialysis perfusion: pharmacological and behavioral studies." Neuroscience **27**(3): 897-904.

Acquas, E., et al. (1998). "Pharmacology of sensory stimulation-evoked increases in frontal cortical acetylcholine release." Neuroscience **85**(1): 73-83.

Becker, C., et al. (1999). "Prevention by 5-HT1A receptor agonists of restraint stress- and yohimbine-induced release of cholecystokinin in the frontal cortex of the freely moving rat." Neuropharmacology **38**(4): 525-532.

Bouchez, G., et al. (2012). "Quantification of extracellular levels of corticosterone in the basolateral amygdaloid complex of freely-moving rats: a dialysis study of circadian variation and stress-induced modulation." Brain Res **1452**: 47-60.

Buffalari, D. M. and A. A. Grace (2009). "Anxiogenic modulation of spontaneous and evoked neuronal activity in the basolateral amygdala." Neuroscience **163**(4): 1069-1077.

Cheng, C. H., et al. (1993). "The profiles of interaction of yohimbine with anxiolytic and putative anxiolytic agents to modify 5-HT release in the frontal cortex of freely-moving rats." Br J Pharmacol **110**(3): 1079-1084.

Dayan, L. and J. P. Finberg (2003). "L-DOPA increases noradrenaline turnover in central and peripheral nervous systems." Neuropharmacology **45**(4): 524-533.

Garcia, A. S., et al. (2004). "Autoreceptor-mediated inhibition of norepinephrine release in rat medial prefrontal cortex is maintained after chronic desipramine treatment." J Neurochem **91**(3): 683-693.

Kushikata, T., et al. (2002). "Alpha-2 adrenoceptor activity affects propofol-induced sleep time." Anesth Analg **94**(5): 1201-1206, table of contents.

Mason, K., et al. (1998). "The anxiogenic agents, yohimbine and FG 7142, disrupt the noradrenergic response to novelty." Pharmacol Biochem Behav **60**(2): 321-327.

Nevo, I., et al. (1996). "Stress- and yohimbine-induced release of cholecystokinin in the frontal cortex of the freely moving rat: prevention by diazepam but not ondansetron." J Neurochem **66**(5): 2041-2049.

Pacak, K., et al. (1993). "In vivo hypothalamic release and synthesis of catecholamines in spontaneously hypertensive rats." Hypertension **22**(4): 467-478.

Shirazi-Southall, S., et al. (2002). "Effects of typical and atypical antipsychotics and receptor selective compounds on acetylcholine efflux in the hippocampus of the rat." Neuropsychopharmacology **26**(5): 583-594.

Sodero, A. O., et al. (2004). "Locus coeruleus activity in perinatally protein-deprived rats: effects of fluoxetine administration." Eur J Pharmacol **503**(1-3): 35-42.

Szemeredi, K., et al. (1991). "Simultaneous measurement of plasma and brain extracellular fluid concentrations of catechols after yohimbine administration in rats." Brain Res **542**(1): 8-14.

**Figure S1.** Extremal changes in local neurotransmitter concentrations (in % relative to basal levels) in response to neuropsychiatric drugs calculated by meta-analysis of in vivo microdialysis experiments.

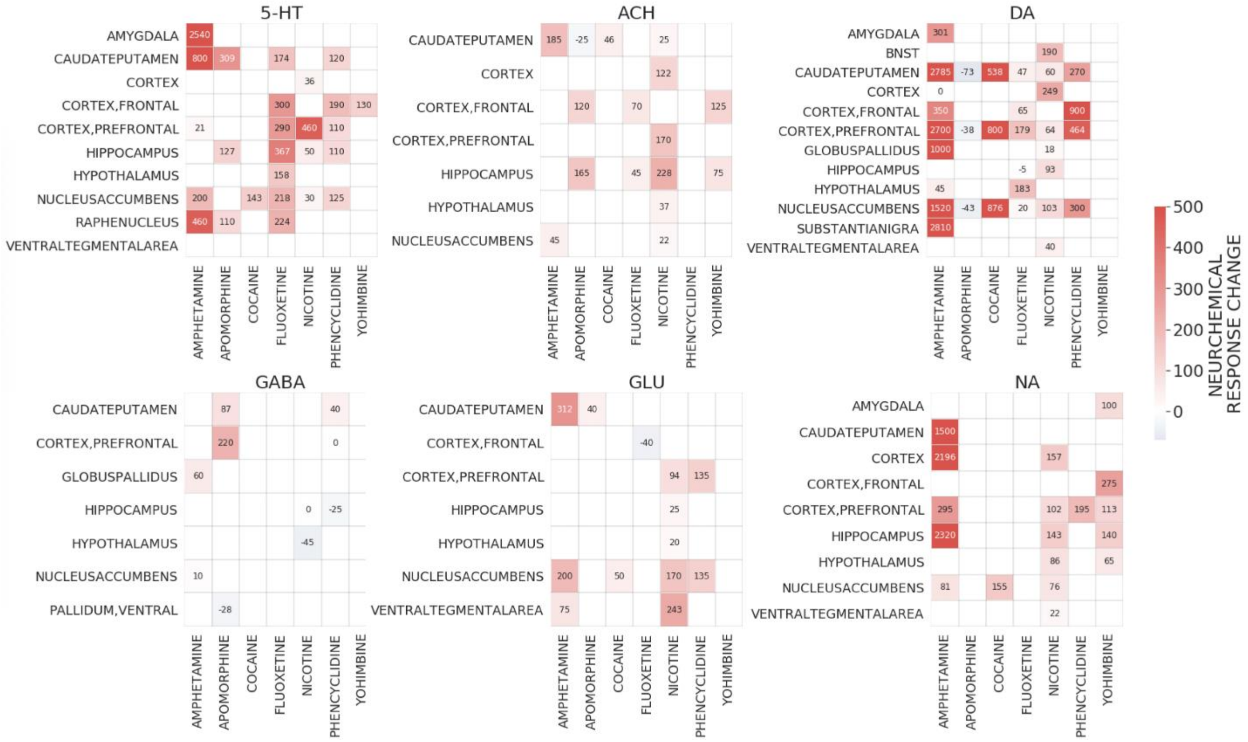

**Figure S2.** Extremal changes in relative cerebral blood volume (% change) in response to neuropsychiatric drugs as measured by phMRI experiments.

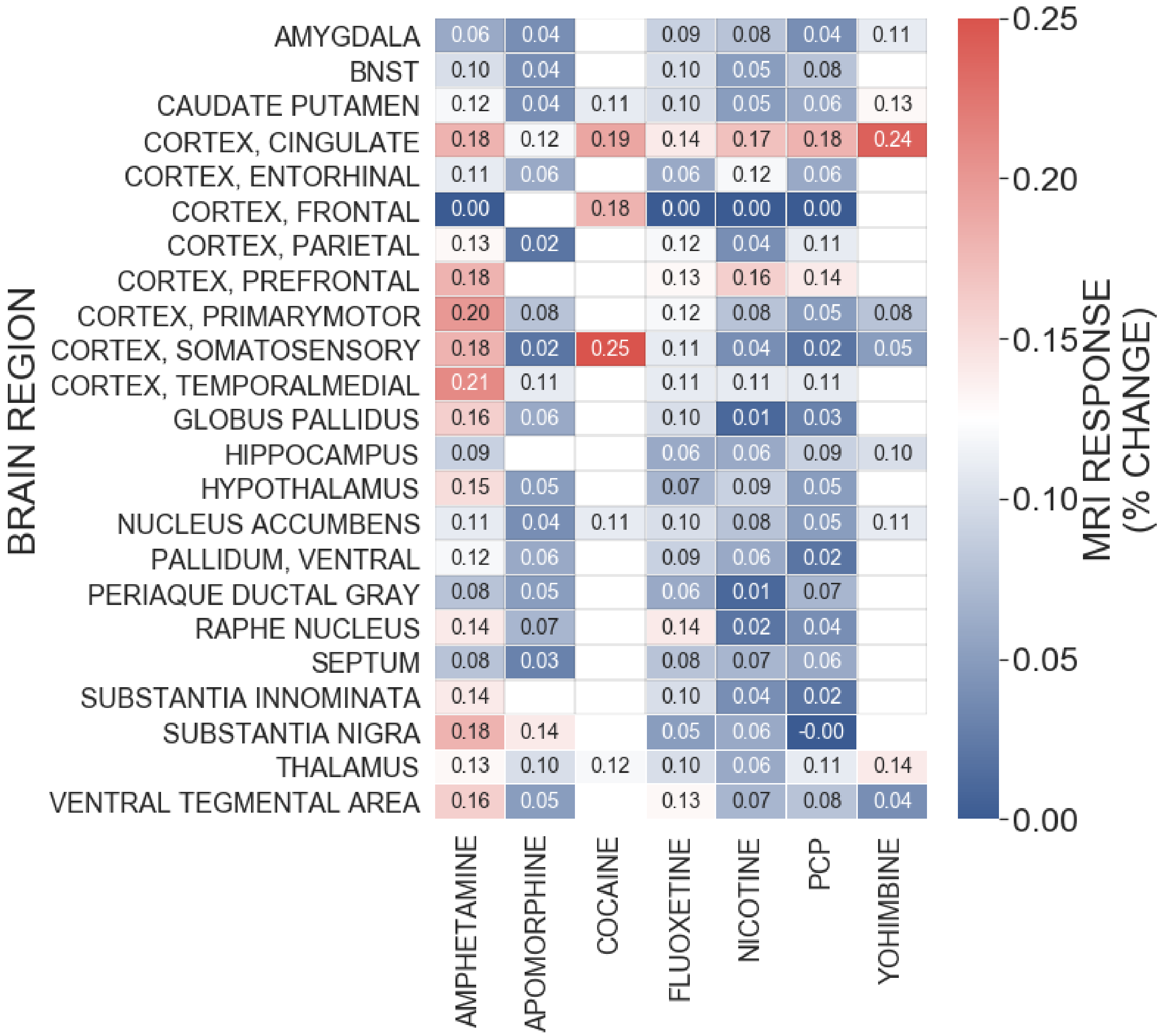

**Figure S3.** Distribution of experimental parameters used in the design of microdialysis experiments. Analogous to the phMRI experiments, the majority of neurochemical data included in our present study relate to experiments conducted in adult, male, Sprague-Dawley rats. It is however critical to note that the quantity of analyte collected by microdialysis (i.e. absolute concentration) often represents only a fraction of the 'true' extracellular neurotransmitter levels. Differences in concentrations of calcium (in mM) of perfusate, flow rate (in µl/min) or membrane surface area (often reported as membrane length (mm) and membrane outer-diameter (µm)) affect the relative recovery, i.e. the ratio between the actual extracellular concentration of an analyte and its dialysate concentration, significantly (Chefer et al., 2009). Therefore, a large number of microdialysis studies on drug effects normalize the drug-induced response to the basal levels measured in the study and report only the normalized (percentages) values.

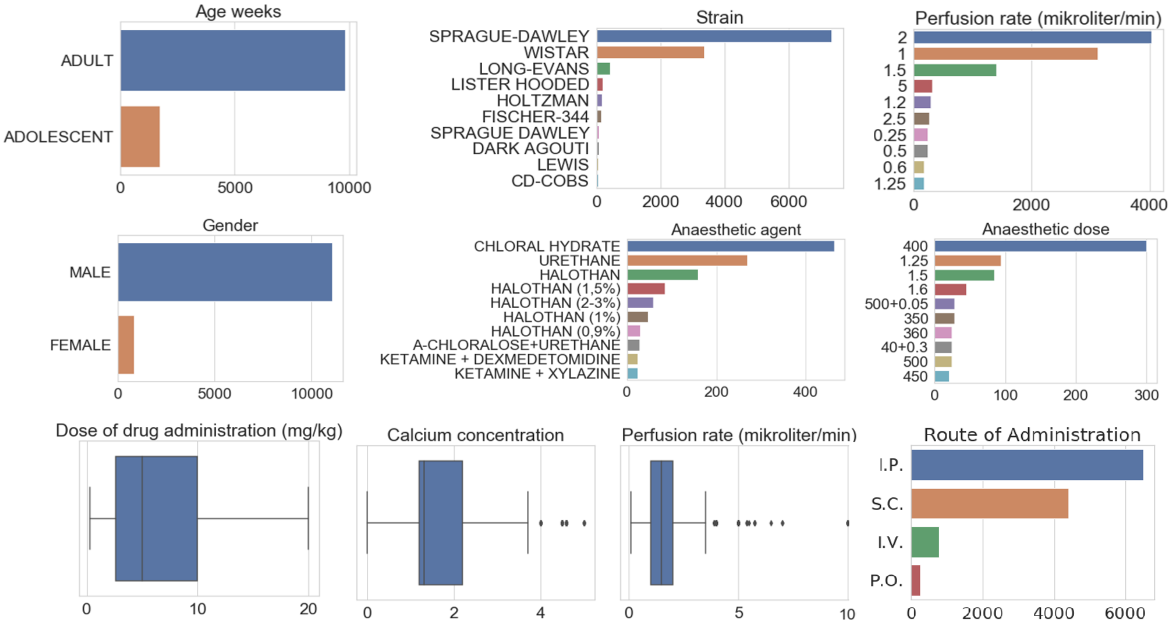

**Figure S4.** Correlation analyses of changes in neurotransmitter levels and rCBV in response to neuropsychiatric drugs in different cortical and subcortical brain areas.

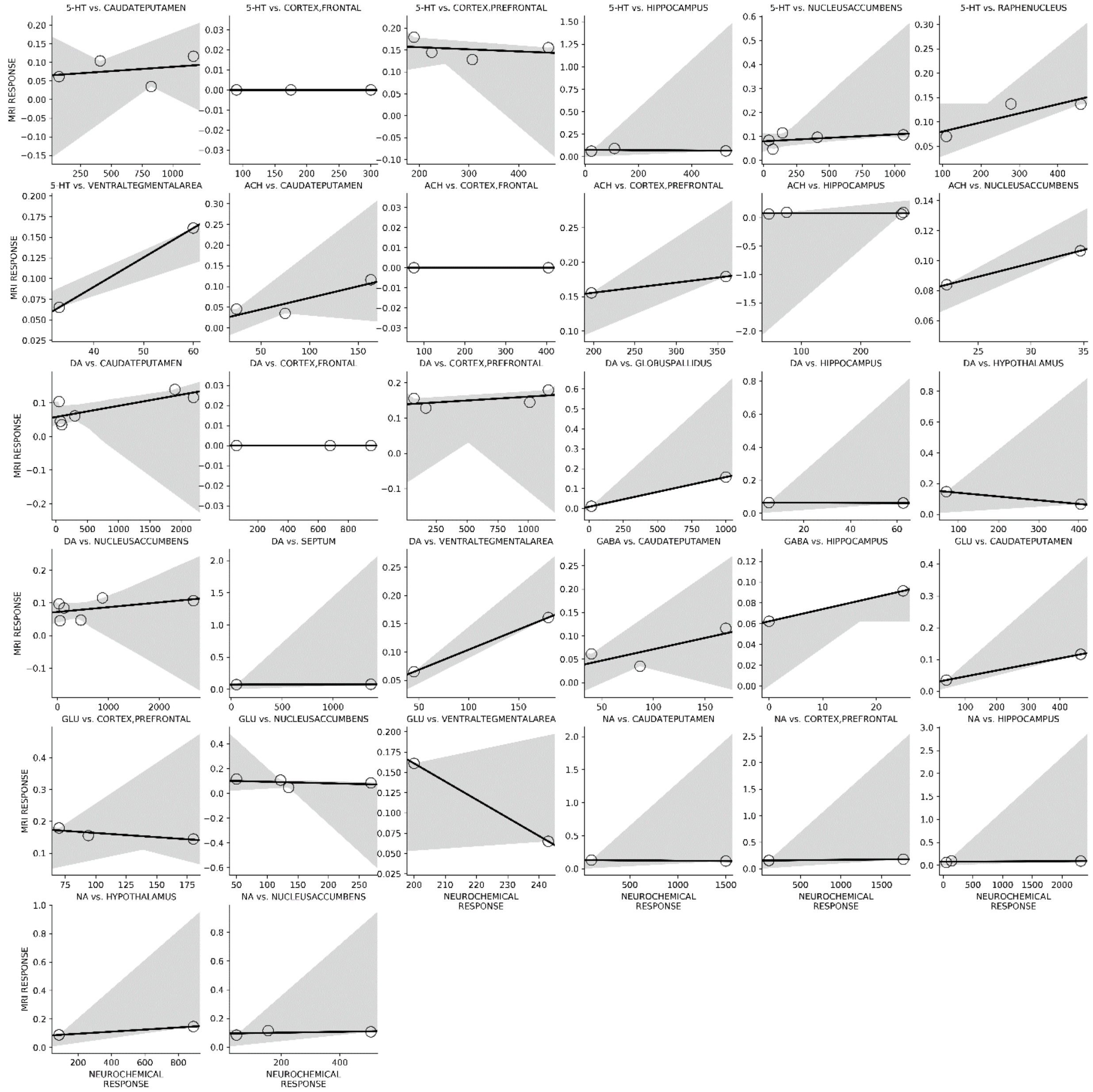

**Figure S5.** Correlation analyses of extremal neurochemical and phMRI response with respect to drugs individually and all together. In addition, we used a OFAT sensitivity analysis (i.e. excluded a drug from an analysis at a time) to investigate whether any drug affects the overall findings qualitatively.

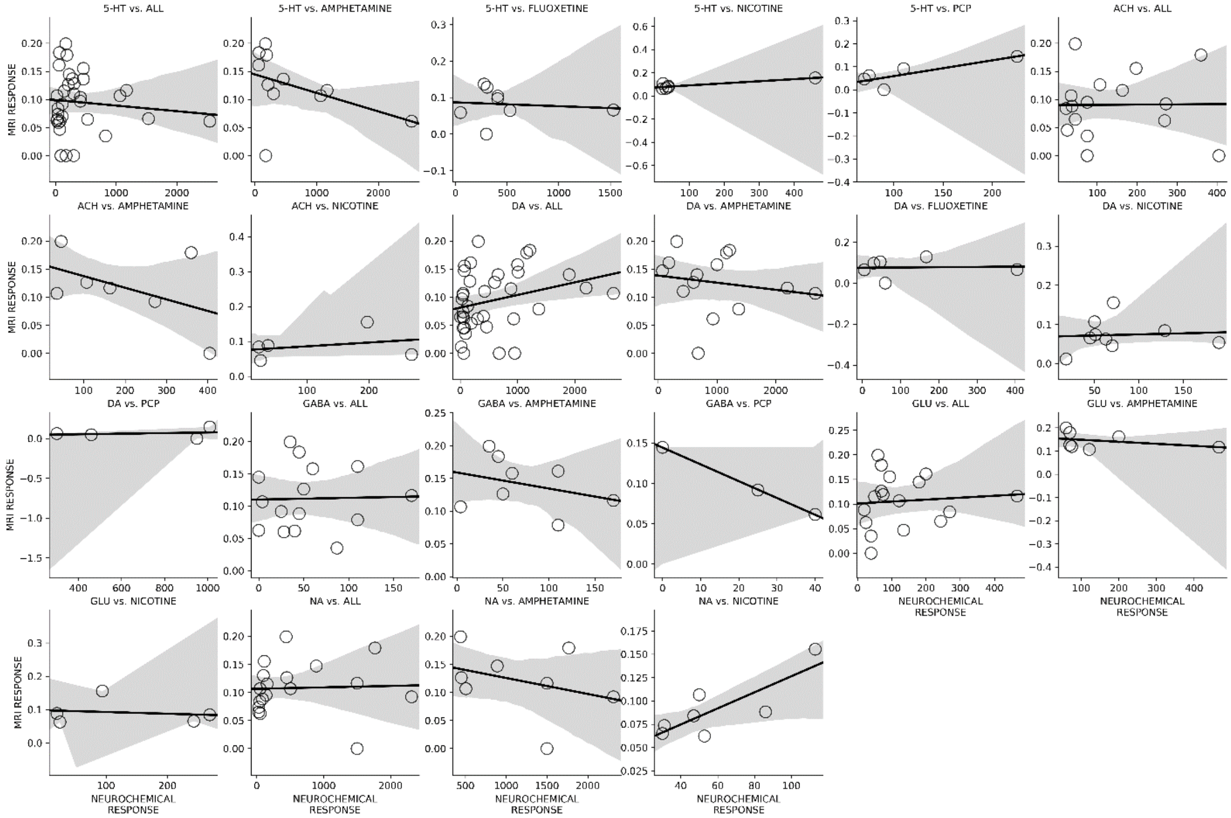

**Figure S5.** Correlation analyses of extremal neurochemical and phMRI response with respect to drugs individually and all together. [continued]

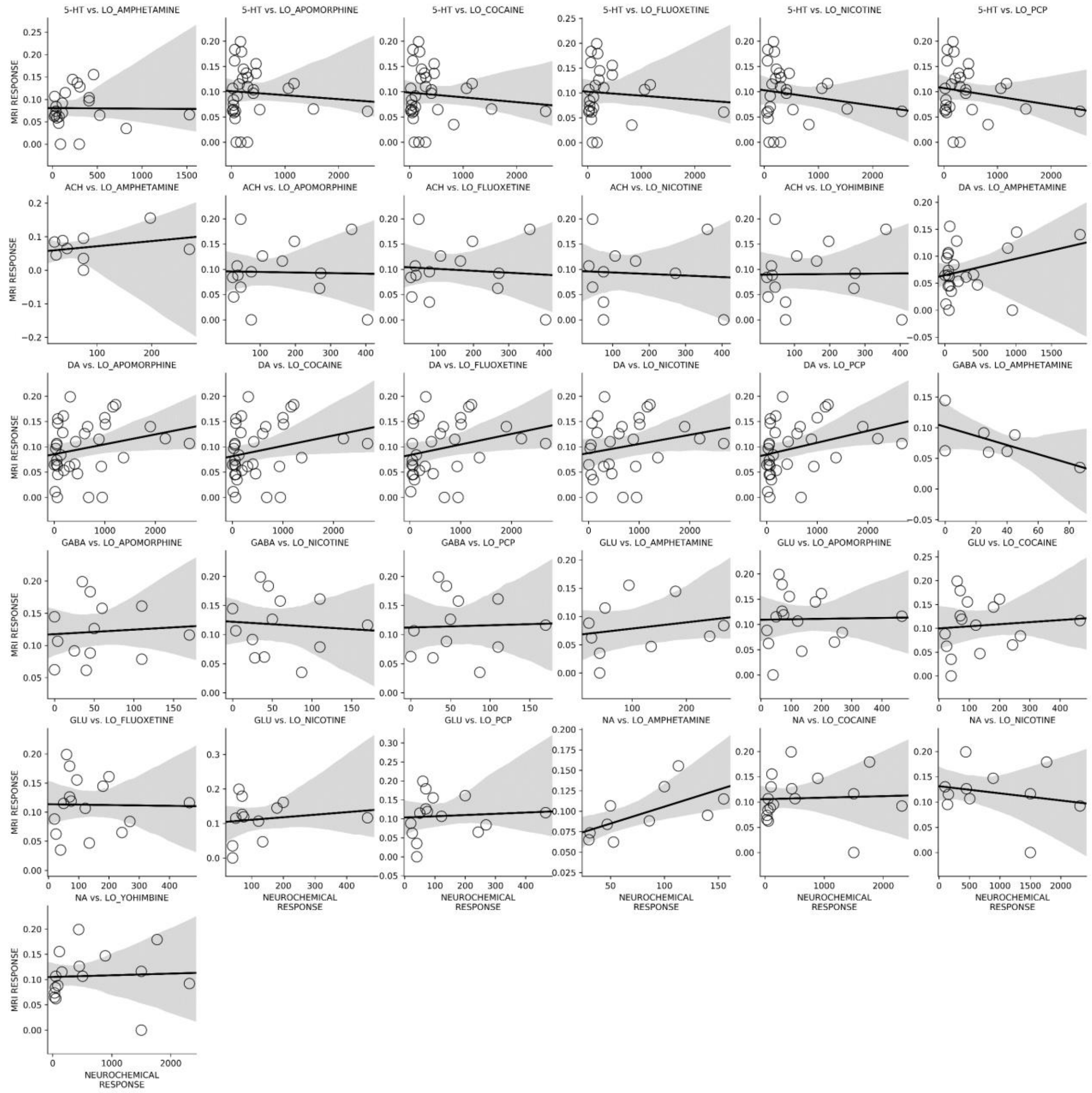

**Figure S5.** Correlation analyses of extremal neurochemical and phMRI response with respect to drugs individually and all together. [continued]

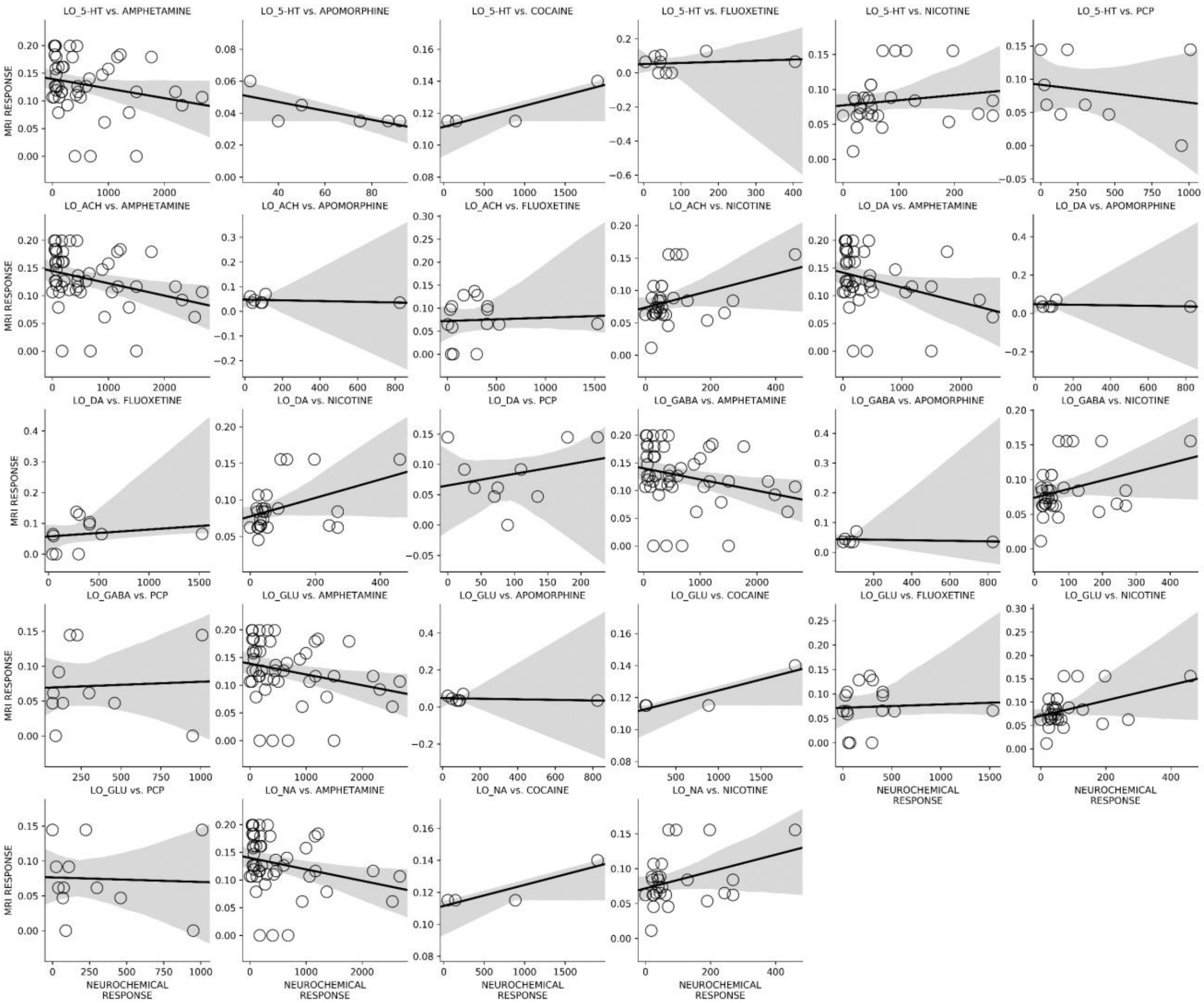

**Figure S6.** Correlation of rCBV and neurochemical peak response times for different neurotransmitters over all cortical and subcortical regions of interest. The bubble size relates to the number of observations.

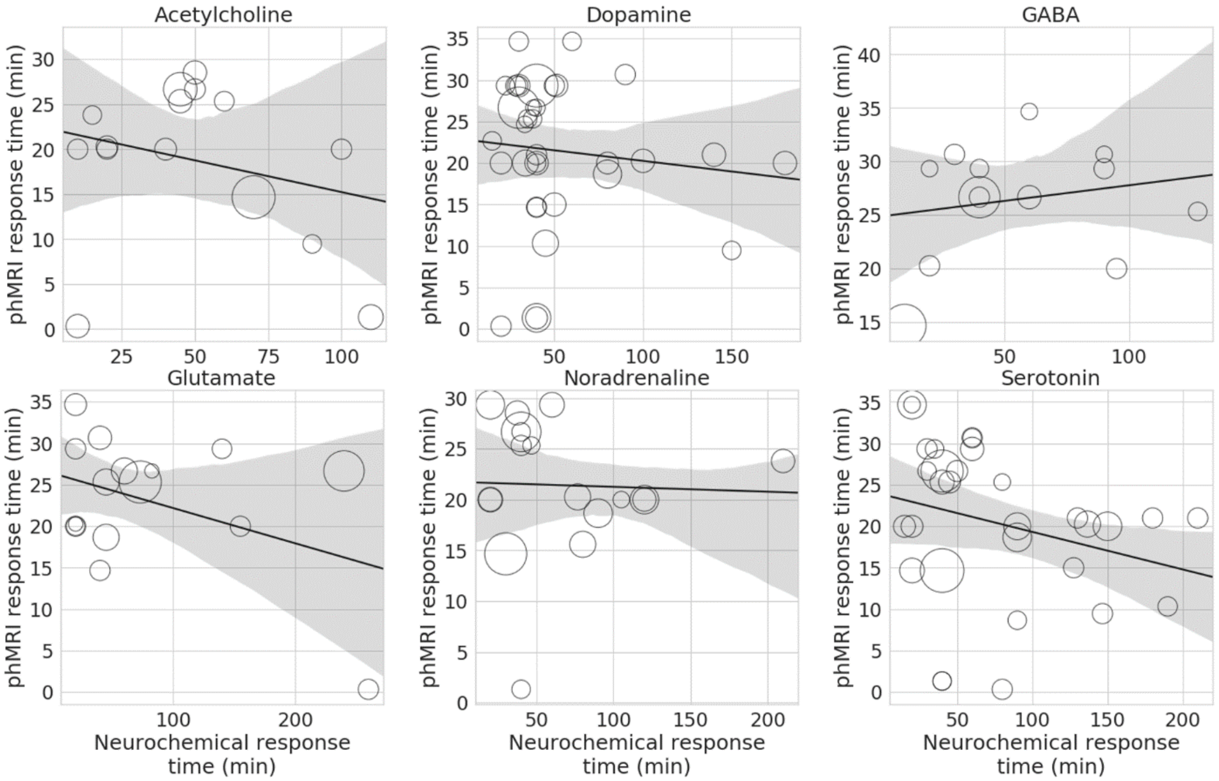

**Table S1.** The distribution of dose of systemically administered neuropsychiatric drugs in relation to the dynamics of neurotransmitter response as measured by microdialysis experiments.

| ***Compound*** | ***Dose of drug (mg/kg)*** | | | | | ***Peak Time (min)*** | | | ***Number of rats*** |
|---|---|---|---|---|---|---|---|---|---|
| | **Average** | **Standard Dev.** | **Median** | **Min** | **Max** | **Average** | **Standard Dev.** | **Median** | |
| *Amphetamine* | 2.80 | 10.51 | 1.5 | 0.03 | 256 | 50.55 | 51.93 | 40 | 12861 |
| *Apomorphine* | 0.62 | 0.71 | 0.5 | 0.05 | 3 | 52.25 | 27.37 | 45 | 446 |
| *Cocaine* | 11.64 | 8.09 | 10 | 0.083 | 40 | 30.55 | 17.28 | 20 | 3280 |
| *Fluoxetine* | 10.08 | 6.21 | 10 | 0.25 | 40 | 98.10 | 65.94 | 80 | 1790 |
| *Nicotine* | 5.89 | 22.04 | 0.4 | 0.04 | 135 | 61.69 | 52.15 | 40 | 3181 |
| *PCP* | 7.10 | 5.12 | 5 | 0.31 | 20 | 61.15 | 47.00 | 40 | 444 |
| *Yohimbine* | 1.98 | 1.49 | 1 | 0.1 | 5 | 63.50 | 55.23 | 40 | 122 |

**Table S2.** Brain regions included in the analysis of common neurochemical and functional response patterns resulting from consistent convergence of stereotaxical MRI brain template ontology and the ontology used in the neurochemical connectome of the rat brain.

| ***Ontology*** |
|---:|
| *nucleus accumbens* |
| *amygdala* |
| *BNST* |
| *caudate putamen* |
| *cortex, cingulate* |
| *cortex, entorhinal* |
| *cortex, frontal* |
| *cortex, prefrontal* |
| *cortex, primary motor* |
| *cortex, parietal* |
| *cortex, somatosensory* |
| *cortex, temporal medial* |
| *globus pallidus* |
| *hippocampus* |
| *periaqueductal gray* |
| *raphe nucleus* |
| *septum* |
| *substantia innominata* |
| *substantia nigra* |
| *thalamus* |
| *pallidum, ventral* |
| *ventral tegmental area* |

**Table S3.** Diversity in the application of anesthesia within 105 in vivo microdialysis experiments (n=2,114).

| ANESTHETIC AGENT | FREQUENCY | RANGE OF APPLIED DOSAGE |
|---|---|---|
| **CHLORAL HYDRATE** | 17.5% | Not reported |
| **HALOTHANE** | 34.5% | 0.9-3% |
| **ISOFLURANE** | 2.7% | 1-4% |
| **KETAMINE (+ MEDETOMIDINE OR XYLAZINE)** | 7.9% | 40-100 mg/kg |
| **PENTOBARBITAL** | 1.8% | Not reported |
| **URETHANE** | 25.8% | Not reported |

**Table S4.** Detailed results of OFAT-sensitivity analysis with respect to neuropsychiatric drugs. LO_drug denotes that the drug were left out the analysis. Averages per-neurotransmitter or per-brain region are shown in bold.

| *NEUROTRANSMITTER BRAIN REGION LEAVE OUT drug* | *SPEARMAN RANK CORRELATION* | *PEARSON CORRELATION* | *NUMBER OF OBSERVATIONS* |
|---|---|---|---|
| ***5-HT*** | **0.3** | **0.2** | **3.0** |
| ***CAUDATEPUTAMEN*** | **0.4** | **0.3** | **3.0** |
| *LO_Amphetamine* | -0.5 | -0.4 | 3.0 |
| *LO_Apomorphine* | 1.0 | 0.9 | 3.0 |
| *LO_Fluoxetine* | 0.5 | 0.5 | 3.0 |
| *LO_PCP* | 0.5 | 0.1 | 3.0 |
| ***CORTEX,PREFRONTAL*** | **-0.4** | **-0.3** | **3.0** |
| *LO_Amphetamine* | 0.5 | 0.5 | 3.0 |
| *LO_Fluoxetine* | -0.5 | -0.3 | 3.0 |
| *LO_Nicotine* | -1.0 | -0.9 | 3.0 |
| *LO_PCP* | -0.5 | -0.4 | 3.0 |
| ***HIPPOCAMPUS*** | **0.4** | **0.2** | **2.3** |
| *LO_Apomorphine* | 0.5 | -0.3 | 3.0 |
| *LO_Fluoxetine* | 1.0 | 1.0 | 2.0 |
| *LO_Nicotine* | -1.0 | -1.0 | 2.0 |
| *LO_PCP* | 1.0 | 1.0 | 2.0 |
| ***NUCLEUSACCUMBENS*** | **0.6** | **0.5** | **4.0** |
| *LO_Amphetamine* | 0.6 | 0.4 | 4.0 |
| *LO_Cocaine* | 0.8 | 0.7 | 4.0 |
| *LO_Fluoxetine* | 0.6 | 0.5 | 4.0 |
| *LO_Nicotine* | 0.4 | 0.5 | 4.0 |
| *LO_PCP* | 0.4 | 0.3 | 4.0 |
| ***RAPHENUCLEUS*** | **0.3** | **0.3** | **2.0** |
| *LO_Amphetamine* | 1.0 | 1.0 | 2.0 |
| *LO_Apomorphine* | -1.0 | -1.0 | 2.0 |
| *LO_Fluoxetine* | 1.0 | 1.0 | 2.0 |
| ***ACH*** | **0.1** | **0.1** | **2.8** |
| ***CAUDATEPUTAMEN*** | **0.3** | **0.3** | **2.0** |
| *LO_Amphetamine* | -1.0 | -1.0 | 2.0 |
| *LO_Apomorphine* | 1.0 | 1.0 | 2.0 |
| *LO_Nicotine* | 1.0 | 1.0 | 2.0 |
| ***HIPPOCAMPUS*** | **0.0** | **0.0** | **3.2** |
| *LO_Amphetamine* | -0.5 | -0.4 | 3.0 |
| *LO_Apomorphine* | 0.0 | 0.0 | 4.0 |
| *LO_Fluoxetine* | -0.5 | -0.6 | 3.0 |
| *LO_Nicotine* | 0.5 | 0.5 | 3.0 |
| *LO_Yohimbine* | 0.5 | 0.4 | 3.0 |
| ***DA*** | **0.5** | **0.5** | **4.3** |
| ***CAUDATEPUTAMEN*** | **0.5** | **0.5** | **5.0** |
| *LO_Amphetamine* | 0.3 | 0.8 | 5.0 |
| *LO_Apomorphine* | 0.6 | 0.3 | 5.0 |
| *LO_Cocaine* | 0.3 | 0.7 | 5.0 |
| *LO_Fluoxetine* | 0.8 | 0.5 | 5.0 |
| *LO_Nicotine* | 0.6 | 0.4 | 5.0 |
| *LO_PCP* | 0.5 | 0.4 | 5.0 |
| ***CORTEX,PREFRONTAL*** | **0.4** | **0.5** | **3.3** |
| *LO_Amphetamine* | -0.5 | 0.0 | 3.0 |
| *LO_Apomorphine* | 0.4 | 0.6 | 4.0 |
| *LO_Cocaine* | 0.4 | 0.6 | 4.0 |

| | | | |
|---|---|---|---|
| *LO_Fluoxetine* | 0.5 | 0.3 | 3.0 |
| *LO_Nicotine* | 1.0 | 0.8 | 3.0 |
| *LO_PCP* | 0.5 | 0.8 | 3.0 |
| ***NUCLEUSACCUMBENS*** | **0.5** | **0.5** | **5.0** |
| *LO_Amphetamine* | 0.3 | 0.4 | 5.0 |
| *LO_Apomorphine* | 0.5 | 0.4 | 5.0 |
| *LO_Cocaine* | 0.3 | 0.5 | 5.0 |
| *LO_Fluoxetine* | 0.8 | 0.6 | 5.0 |
| *LO_Nicotine* | 0.6 | 0.5 | 5.0 |
| *LO_PCP* | 0.6 | 0.5 | 5.0 |
| ***SEPTUM*** | **1.0** | **1.0** | **2.0** |
| *LO_Cocaine* | 1.0 | 1.0 | 2.0 |
| ***GABA*** | **0.3** | **0.3** | **2.0** |
| ***CAUDATEPUTAMEN*** | **0.3** | **0.3** | **2.0** |
| *LO_Amphetamine* | -1.0 | -1.0 | 2.0 |
| *LO_Apomorphine* | 1.0 | 1.0 | 2.0 |
| *LO_PCP* | 1.0 | 1.0 | 2.0 |
| ***GLU*** | **-0.9** | **-0.7** | **2.6** |
| ***CORTEX,PREFRONTAL*** | **-1.0** | **-1.0** | **2.0** |
| *LO_Amphetamine* | -1.0 | -1.0 | 2.0 |
| *LO_Nicotine* | -1.0 | -1.0 | 2.0 |
| *LO_PCP* | -1.0 | -1.0 | 2.0 |
| ***NUCLEUSACCUMBENS*** | **-0.8** | **-0.5** | **3.0** |
| *LO_Amphetamine* | -0.5 | -0.3 | 3.0 |
| *LO_Cocaine* | -0.5 | 0.1 | 3.0 |
| *LO_Nicotine* | -1.0 | -0.7 | 3.0 |
| *LO_PCP* | -1.0 | -1.0 | 3.0 |
| ***NA_TRANSMITTER*** | **0.5** | **0.5** | **2.0** |
| ***CORTEX,PREFRONTAL*** | **1.0** | **1.0** | **2.0** |
| *LO_Yohimbine* | 1.0 | 1.0 | 2.0 |
| ***HIPPOCAMPUS*** | **0.3** | **0.3** | **2.0** |
| *LO_Amphetamine* | 1.0 | 1.0 | 2.0 |
| *LO_Nicotine* | -1.0 | -1.0 | 2.0 |
| *LO_Yohimbine* | 1.0 | 1.0 | 2.0 |
| ***HYPOTHALAMUS*** | **1.0** | **1.0** | **2.0** |
| *LO_Yohimbine* | 1.0 | 1.0 | 2.0 |
| ***NUCLEUSACCUMBENS*** | **0.3** | **0.3** | **2.0** |
| *LO_Amphetamine* | 1.0 | 1.0 | 2.0 |
| *LO_Cocaine* | 1.0 | 1.0 | 2.0 |
| *LO_Nicotine* | -1.0 | -1.0 | 2.0 |

**Table S5**. Detailed results of correlation and sensitivity analysis grouped by neurotransmitters and neuropsychiatric drugs. Averages per-neurotransmitter are shown in bold. *ALL* refers to the calculation of correlations for all drugs taken together.

| *NEUROTRANSMITTER COMPOUND* | *SPEARMAN RANK CORRELATION* | *PEARSON CORRELATION* | *NUMBER OF OBSERVATIONS* |
|---|---|---|---|
| ***5-HT*** | **-0.15** | **0.01** | **66** |
| *ALL* | 0.10 | -0.09 | 33 |
| *Amphetamine* | -0.61 | -0.43 | 11 |
| *Apomorphine* | -1.00 | -1.00 | 2 |
| *Cocaine* | | | 1 |
| *Fluoxetine* | 0.02 | -0.11 | 8 |
| *Nicotine* | 0.64 | 0.89 | 6 |
| *PCP* | 0.70 | 0.82 | 5 |
| *Yohimbine* | | | 0 |
| ***ACH*** | **-1.10** | **-0.29** | **32** |
| *ALL* | 0.09 | 0.01 | 16 |
| *Amphetamine* | -0.39 | -0.48 | 7 |
| *Apomorphine* | | | 1 |
| *Cocaine* | | | 0 |
| *Fluoxetine* | -1.00 | -1.00 | 2 |
| *Nicotine* | 0.20 | 0.30 | 5 |
| *PCP* | | | 0 |
| *Yohimbine* | | | 1 |
| ***DA*** | **-1.43** | **-0.23** | **74** |
| *ALL* | 0.24 | 0.09 | 37 |
| *Amphetamine* | -0.28 | -0.06 | 14 |
| *Apomorphine* | -1.00 | -1.00 | 2 |
| *Cocaine* | -1.00 | -1.00 | 2 |
| *Fluoxetine* | 0.20 | 0.05 | 6 |
| *Nicotine* | 0.20 | 0.07 | 9 |
| *PCP* | 0.20 | 0.22 | 4 |
| *Yohimbine* | | | 0 |
| ***GABA*** | **-1.20** | **-0.26** | **30** |
| *ALL* | 0.10 | 0.02 | 15 |
| *Amphetamine* | -0.30 | -0.32 | 8 |
| *Apomorphine* | -1.00 | -1.00 | 2 |
| *Cocaine* | | | 0 |
| *Fluoxetine* | | | 0 |
| *Nicotine* | 1.00 | 1.00 | 2 |
| *PCP* | -1.00 | -1.00 | 3 |
| *Yohimbine* | | | 0 |
| ***GLU*** | **0.48** | **0.15** | **34** |
| *ALL* | 0.27 | 0.12 | 17 |
| *Amphetamine* | -0.68 | -0.36 | 7 |
| *Apomorphine* | | | 1 |
| *Cocaine* | | | 1 |
| *Fluoxetine* | | | 1 |
| *Nicotine* | -0.10 | -0.16 | 5 |
| *PCP* | 1.00 | 1.00 | 2 |
| *Yohimbine* | | | 0 |
| ***LO_5-HT*** | **-1.63** | **-0.26** | **100** |
| *Amphetamine* | -0.29 | -0.06 | 44 |

| | | | |
|---|---|---|---|
| *Apomorphine* | -0.68 | -0.72 | 6 |
| *Cocaine* | -0.77 | -0.90 | 4 |
| *Fluoxetine* | 0.16 | 0.17 | 9 |
| *Nicotine* | 0.24 | 0.16 | 28 |
| *PCP* | -0.29 | -0.20 | 9 |
| ***LO_ACH*** | **-1.04** | **-0.19** | **101** |
| *Amphetamine* | -0.39 | -0.07 | 48 |
| *Apomorphine* | -0.16 | -0.29 | 7 |
| *Fluoxetine* | 0.19 | 0.06 | 15 |
| *Nicotine* | 0.32 | 0.36 | 29 |
| *Yohimbine* | -1.00 | -1.00 | 2 |
| ***LO_DA*** | **0.51** | **0.06** | **96** |
| *Amphetamine* | -0.37 | -0.33 | 41 |
| *Apomorphine* | -0.07 | -0.29 | 6 |
| *Cocaine* | | | 3 |
| *Fluoxetine* | 0.47 | 0.19 | 11 |
| *Nicotine* | 0.39 | 0.43 | 25 |
| *PCP* | 0.10 | 0.27 | 10 |
| ***LO_GABA*** | **0.21** | **0.03** | **96** |
| *Amphetamine* | -0.36 | -0.05 | 47 |
| *Apomorphine* | 0.10 | -0.21 | 6 |
| *Nicotine* | 0.27 | 0.34 | 32 |
| *PCP* | 0.19 | 0.06 | 11 |
| ***LO_GLU*** | **-0.99** | **-0.14** | **116** |
| *Amphetamine* | -0.32 | -0.05 | 48 |
| *Apomorphine* | -0.26 | -0.31 | 7 |
| *Cocaine* | -0.77 | -0.91 | 4 |
| *Fluoxetine* | 0.19 | 0.06 | 16 |
| *Nicotine* | 0.33 | 0.44 | 29 |
| *PCP* | -0.16 | -0.05 | 12 |
| ***LO_NA*** | **-0.84** | **-0.20** | **79** |
| *Amphetamine* | -0.35 | -0.06 | 47 |
| *Cocaine* | -0.77 | -0.90 | 4 |
| *Nicotine* | 0.28 | 0.36 | 27 |
| *Yohimbine* | | | 1 |
| ***NA*** | **-0.51** | **-0.11** | **36** |
| *ALL* | 0.36 | 0.08 | 18 |
| *Amphetamine* | -0.44 | -0.33 | 8 |
| *Apomorphine* | | | 0 |
| *Cocaine* | | | 1 |
| *Fluoxetine* | | | 0 |
| *Nicotine* | 0.57 | 0.81 | 7 |
| *PCP* | | | 0 |
| *Yohimbine* | -1.00 | -1.00 | 2 |

**Table S6.** Statistical inference of the Pearson correlation analyses for each neurotransmitter system presented by p-values and confidence intervals. LO (left out) refers to OFAT analysis with respect to a neurotransmitter. $CI_{lb}$ and $CI_{ub}$ denotes the lower and upper bounds of the confidence interval.

| NEUROCHEMICAL COMPONENT | COMPOUND | PEARSON CORRELATION | PEARSON CORRELATION PVALUE | $CI_{lb}$ | $CI_{ub}$ | NUMBER OF OBSERVATIONS |
|---|---|---|---|---|---|---|
| **GABA** | PCP | -0.99991 | 0.008606 | 0 | 2 | 3 |
| **LO_GLU** | Nicotine | 0.44313 | 0.016061 | 0.303485 | 0.908519 | 29 |
| **5-HT** | Nicotine | 0.888375 | 0.017995 | 0.012222 | 0.72471 | 6 |
| **NA** | Nicotine | 0.810408 | 0.027065 | 0.029074 | 0.852841 | 7 |
| **LO_DA** | Nicotine | 0.427026 | 0.033256 | 0.296539 | 0.961631 | 25 |
| **LO_DA** | Amphetamine | -0.32532 | 0.037943 | 1.019634 | 1.575383 | 41 |
| **NA** | LO_Amphetamine | 0.629135 | 0.051321 | 0.098386 | 1.000813 | 10 |
| **LO_ACH** | Nicotine | 0.362346 | 0.053399 | 0.356594 | 1.004797 | 29 |
| **LO_GABA** | Nicotine | 0.343473 | 0.054268 | 0.381865 | 1.005931 | 32 |
| **LO_NA** | Nicotine | 0.360948 | 0.064355 | 0.348413 | 1.022097 | 27 |
| **5-HT** | PCP | 0.816909 | 0.091418 | 0.012527 | 1.234032 | 5 |
| **LO_GLU** | Cocaine | 0.90827 | 0.09173 | 0.001906 | 1.415645 | 4 |
| **LO_5-HT** | Cocaine | 0.90038 | 0.09962 | 0.002078 | 1.450828 | 4 |
| **LO_NA** | Cocaine | 0.899822 | 0.100178 | 0.00209 | 1.453169 | 4 |
| **LO_5-HT** | Apomorphine | -0.72071 | 0.106115 | 0.781124 | 1.966793 | 6 |
| **GABA** | LO_Amphetamine | -0.63642 | 0.124355 | 0.776015 | 1.939306 | 7 |
| **DA** | LO_Amphetamine | 0.320812 | 0.135559 | 0.352598 | 1.105318 | 23 |
| **5-HT** | Amphetamine | -0.42733 | 0.189875 | 0.76798 | 1.817615 | 11 |
| **ACH** | Amphetamine | -0.47686 | 0.279268 | 0.569046 | 1.904949 | 7 |
| **5-HT** | LO_PCP | -0.18438 | 0.347599 | 0.797365 | 1.521579 | 28 |
| **LO_5-HT** | Nicotine | 0.16046 | 0.414689 | 0.496599 | 1.226155 | 28 |
| **NA** | Amphetamine | -0.32863 | 0.426748 | 0.510629 | 1.839008 | 8 |
| **GLU** | Amphetamine | -0.35931 | 0.428614 | 0.460191 | 1.87548 | 7 |
| **ACH** | LO_Amphetamine | 0.300074 | 0.432726 | 0.196025 | 1.454654 | 9 |
| **5-HT** | LO_Nicotine | -0.15541 | 0.438909 | 0.761298 | 1.505565 | 27 |
| **GABA** | Amphetamine | -0.31842 | 0.442085 | 0.502009 | 1.835601 | 8 |
| **LO_DA** | PCP | 0.270798 | 0.449191 | 0.230736 | 1.432585 | 10 |
| **LO_GLU** | Apomorphine | -0.30842 | 0.500957 | 0.420847 | 1.861408 | 7 |
| **LO_ACH** | Apomorphine | -0.29173 | 0.525544 | 0.40879 | 1.856602 | 7 |
| **NA** | LO_Nicotine | -0.20856 | 0.538277 | 0.552727 | 1.718539 | 11 |
| **GLU** | LO_Amphetamine | 0.210658 | 0.559096 | 0.258116 | 1.483036 | 10 |
| **LO_DA** | Fluoxetine | 0.193481 | 0.568663 | 0.289148 | 1.459755 | 11 |
| **5-HT** | ALL | -0.10209 | 0.571834 | 0.750023 | 1.43032 | 33 |
| **LO_DA** | Apomorphine | -0.28922 | 0.57826 | 0.317454 | 1.891524 | 6 |
| **5-HT** | LO_Cocaine | -0.09715 | 0.596842 | 0.739635 | 1.431235 | 32 |
| **DA** | LO_Cocaine | 0.09108 | 0.602817 | 0.588173 | 1.249746 | 35 |
| **DA** | ALL | 0.088045 | 0.604331 | 0.599366 | 1.242904 | 37 |
| **LO_5-HT** | PCP | -0.19945 | 0.606904 | 0.464384 | 1.762565 | 9 |
| **ACH** | Nicotine | 0.298289 | 0.625917 | 0.065403 | 1.792554 | 5 |
| **GLU** | LO_Nicotine | 0.149177 | 0.643562 | 0.333944 | 1.464491 | 12 |
| **DA** | LO_PCP | 0.083324 | 0.644793 | 0.585232 | 1.267641 | 33 |
| **LO_ACH** | Amphetamine | -0.0676 | 0.648021 | 0.779223 | 1.345105 | 48 |
| **DA** | LO_Apomorphine | 0.078074 | 0.655744 | 0.59911 | 1.261989 | 35 |
| **LO_5-HT** | Fluoxetine | 0.17131 | 0.659433 | 0.249907 | 1.556077 | 9 |
| **5-HT** | LO_Apomorphine | -0.08176 | 0.661929 | 0.719288 | 1.423822 | 31 |
| **DA** | LO_Fluoxetine | 0.079886 | 0.66924 | 0.577727 | 1.28245 | 31 |
| **LO_GABA** | Apomorphine | -0.21213 | 0.686574 | 0.275914 | 1.873341 | 6 |
| **5-HT** | LO_Fluoxetine | -0.08387 | 0.690215 | 0.678066 | 1.463633 | 25 |
| **LO_NA** | Amphetamine | -0.05884 | 0.6944 | 0.767753 | 1.340261 | 47 |
| **LO_5-HT** | Amphetamine | -0.05682 | 0.71409 | 0.755824 | 1.347836 | 44 |
| **GLU** | LO_Cocaine | 0.093354 | 0.730937 | 0.436994 | 1.421874 | 16 |
| **DA** | LO_Nicotine | 0.067691 | 0.732164 | 0.570089 | 1.313298 | 28 |
| **LO_GABA** | Amphetamine | -0.05038 | 0.736623 | 0.759741 | 1.332737 | 47 |
| **LO_GLU** | Amphetamine | -0.04942 | 0.738686 | 0.761946 | 1.328939 | 48 |
| **GLU** | ALL | 0.084534 | 0.747021 | 0.456889 | 1.412887 | 17 |
| **ACH** | LO_Fluoxetine | -0.08917 | 0.761783 | 0.536669 | 1.591752 | 14 |
| **DA** | PCP | 0.220106 | 0.779894 | 0.025049 | 1.939784 | 4 |
| **GABA** | LO_Nicotine | -0.08122 | 0.791969 | 0.508224 | 1.605122 | 13 |
| **GLU** | LO_PCP | 0.0743 | 0.792427 | 0.434944 | 1.455292 | 15 |
| **GLU** | Nicotine | -0.16207 | 0.794548 | 0.159643 | 1.91369 | 5 |
| **GABA** | LO_Apomorphine | 0.077151 | 0.80219 | 0.397477 | 1.494871 | 13 |
| **5-HT** | Fluoxetine | -0.10616 | 0.802441 | 0.353093 | 1.754398 | 8 |
| **LO_GLU** | Fluoxetine | 0.057082 | 0.833685 | 0.462424 | 1.451396 | 16 |
| **LO_ACH** | Fluoxetine | 0.05877 | 0.835193 | 0.445656 | 1.46757 | 15 |
| **DA** | Amphetamine | -0.06066 | 0.836791 | 0.514454 | 1.572805 | 14 |
| **NA** | LO_Yohimbine | 0.050727 | 0.852 | 0.466971 | 1.456456 | 16 |
| **DA** | Nicotine | 0.071743 | 0.854472 | 0.297602 | 1.622015 | 9 |

| | | | | | | |
|---|---|---|---|---|---|---|
| **ACH** | LO_Nicotine | -0.06255 | 0.855034 | 0.441727 | 1.638468 | 11 |
| **NA** | LO_Cocaine | 0.045449 | 0.862489 | 0.485153 | 1.444915 | 17 |
| **LO_GABA** | PCP | 0.056334 | 0.869326 | 0.365241 | 1.562551 | 11 |
| **NA** | ALL | 0.039428 | 0.876563 | 0.502854 | 1.435458 | 18 |
| **LO_GLU** | PCP | -0.04534 | 0.888731 | 0.457316 | 1.603536 | 12 |
| **GABA** | LO_PCP | 0.036273 | 0.91089 | 0.402268 | 1.549058 | 12 |
| **ACH** | LO_Apomorphine | -0.02573 | 0.927479 | 0.506968 | 1.530993 | 15 |
| **GABA** | ALL | 0.024851 | 0.929949 | 0.469638 | 1.493697 | 15 |
| **DA** | Fluoxetine | 0.045853 | 0.931268 | 0.173347 | 1.795303 | 6 |
| **GLU** | LO_Apomorphine | 0.019835 | 0.941876 | 0.489479 | 1.480595 | 16 |
| **GLU** | LO_Fluoxetine | -0.01699 | 0.950199 | 0.51722 | 1.508415 | 16 |
| **5-HT** | LO_Amphetamine | -0.01075 | 0.962119 | 0.587274 | 1.43041 | 22 |
| **ACH** | LO_Yohimbine | 0.012843 | 0.963766 | 0.478326 | 1.502727 | 15 |
| **ACH** | ALL | 0.009964 | 0.970787 | 0.496816 | 1.488153 | 16 |

**Table S7.** Statistical inference of the Pearson correlation analyses for each neurotransmitter system and brain region presented by p-values and confidence intervals.

| NEUROCHEMICAL COMPONENT | BRAIN REGION | PEARSON CORRELATION | PEARSON CORRELATION PVALUE | $CI_{lb}$ | $CI_{ub}$ | NUMBER OF OBSERVATIONS |
|---|---|---|---|---|---|---|
| **ACH** | CORTEX,FRONTAL | | | | | 2 |
| **5-HT** | CORTEX,FRONTAL | | | | | 3 |
| **DA** | CORTEX,FRONTAL | | | | | 3 |
| **GLU** | CAUDATEPUTAMEN | 1 | | | | 2 |
| **ACH** | CORTEX,PREFRONTAL | 1 | | | | 2 |
| **NA** | CORTEX,PREFRONTAL | 1 | | | | 2 |
| **DA** | GLOBUSPALLIDUS | 1 | | | | 2 |
| **NA** | HYPOTHALAMUS | 1 | | | | 2 |
| **ACH** | NUCLEUSACCUMBENS | 1 | | | | 2 |
| **DA** | SEPTUM | 1 | | | | 2 |
| **5-HT** | VENTRALTEGMENTALAREA | 1 | | | | 2 |
| **DA** | VENTRALTEGMENTALAREA | 1 | | | | 2 |
| **GABA** | HIPPOCAMPUS | 1 | | | | 2 |
| **ACH** | CAUDATEPUTAMEN | 0.885667 | 0.307403 | 0.114333 | 2 | 3 |
| **5-HT** | RAPHENUCLEUS | 0.851426 | 0.351476 | 0.148574 | 2 | 3 |
| **GABA** | CAUDATEPUTAMEN | 0.771873 | 0.43864 | 0.228127 | 2 | 3 |
| **DA** | CORTEX,PREFRONTAL | 0.574053 | 0.425947 | 0.425947 | 1.863366 | 4 |
| **DA** | NUCLEUSACCUMBENS | 0.496721 | 0.316196 | 0.503279 | 1.527476 | 6 |
| **NA** | NUCLEUSACCUMBENS | 0.466223 | 0.691229 | 0.533777 | 2 | 3 |
| **5-HT** | NUCLEUSACCUMBENS | 0.461576 | 0.433895 | 0.538424 | 1.709706 | 5 |
| **NA** | HIPPOCAMPUS | 0.459994 | 0.695703 | 0.540006 | 2 | 3 |
| **DA** | CAUDATEPUTAMEN | 0.399465 | 0.432674 | 0.600535 | 1.609781 | 6 |
| **5-HT** | CAUDATEPUTAMEN | 0.293142 | 0.706858 | 0.706858 | 1.929942 | 4 |
| **ACH** | HIPPOCAMPUS | -0.00681 | 0.993195 | 1.006805 | 1.961603 | 4 |
| **5-HT** | CORTEX,PREFRONTAL | -0.26915 | 0.730855 | 1.269145 | 1.977405 | 4 |
| **5-HT** | HIPPOCAMPUS | -0.28671 | 0.814875 | 1.286712 | 2 | 3 |
| **GLU** | NUCLEUSACCUMBENS | -0.37008 | 0.62992 | 1.37008 | 1.981919 | 4 |
| **GLU** | CORTEX,PREFRONTAL | -0.86391 | 0.336023 | 1.863905 | 2 | 3 |
| **NA** | CAUDATEPUTAMEN | -1 | | | | 2 |
| **DA** | HIPPOCAMPUS | -1 | | | | 2 |
| **DA** | HYPOTHALAMUS | -1 | | | | 2 |
| **GLU** | VENTRALTEGMENTALAREA | -1 | | | | 2 |

**Table S8.** Statistical distribution of phMRI peak response time to neuropsychiatric drugs over all cortical and subcortical regions of interest.

| PEAK RESPONSE (MINS) | NICOTINE | PCP | AMPHETAMINE | FLUOXETINE | YOHIMBINE | APOMORPHINE | COCAINE |
|---|---|---|---|---|---|---|---|
| **MIN** | 19 | 5 | 13 | 1 | 3 | 4.5 | 17 |
| **MAX** | 39 | 56 | 29 | 64 | 7 | 5.5 | 22 |
| **MEDIAN** | 20 | 35 | 28 | 31 | 5 | 5.5 | 18.5 |
| **MEAN** | 20 | 36 | 25 | 60 | 5 | 5.3 | 19.1 |
